# DARTS: Multi-year database of AI-detected retrogressive thaw slumps in the circum-arctic permafrost region

**DOI:** 10.1038/s41597-025-05810-2

**Published:** 2025-08-29

**Authors:** Ingmar Nitze, Konrad Heidler, Nina Nesterova, Jonas Küpper, Emma Schütt, Tobias Hölzer, Sophia Barth, Mark J. Lara, Anna K. Liljedahl, Guido Grosse

**Affiliations:** 1 Permafrost Research Section, Alfred Wegener Institute Helmholtz Centre for Polar and Marine Research, 14473 Potsdam, Germany; 2 Chair of Data Science in Earth Observation (SiPEO), Department of Aerospace and Geodesy, TUM School of Engineering and Design, Technical University of Munich (TUM), 80333 Munich, Germany; 3 Institute of Geosciences, University of Potsdam, 14469 Potsdam, Germany; 4 Computing and Data Centre, Alfred Wegener Institute Helmholtz Centre for Polar and Marine Research, 27570 Bremerhaven, Germany; 5 Department of Plant Biology, University of Illinois at Urbana-Champaign, 61801 Urbana, Illinois USA; 6 Department of Geography, University of Illinois at Urbana-Champaign, 61801 Urbana, Illinois USA; 7 Woodwell Climate Research Center, 02540 Falmouth, Massachusetts USA

**Keywords:** Natural hazards, Cryospheric science

## Abstract

Retrogressive Thaw Slumps (RTS) are widespread mass-wasting hillslope failures triggered by thawing permafrost. While regional studies have provided insights into the spatial distribution and dynamics of RTS, a consistent and unbiased quantification and monitoring remains unsolved at pan-arctic scales. We present the Database of AI-detected Arctic RTS footprints (DARTS), comprising ~43,000 individual footprints of active RTS or active areas within larger RTS landforms. DARTS spans ~1.6 million km^2^ from 2018–2023, with at least annual coverage from 2021–2023 across a ~900,000 km^2^ region. The database is freely available in two processing levels: sub-annual and annually aggregated polygon footprints including spatial and tabular metadata. DARTS uses a highly automated workflow based on deep learning segmentation of PlanetScope multi-spectral satellite imagery (3–5 m resolution) and elevation data. Validation against different regional RTS datasets yielded F1 scores ranging from 0.263 to 0.700, with higher accuracy in areas of intense RTS activity. DARTS provides a valuable resource for systematically mapping, quantifying, and analyzing active hillslope thermokarst distribution and changes over time across the circum-arctic permafrost region.

## Background & Summary

**Fig. 1:**
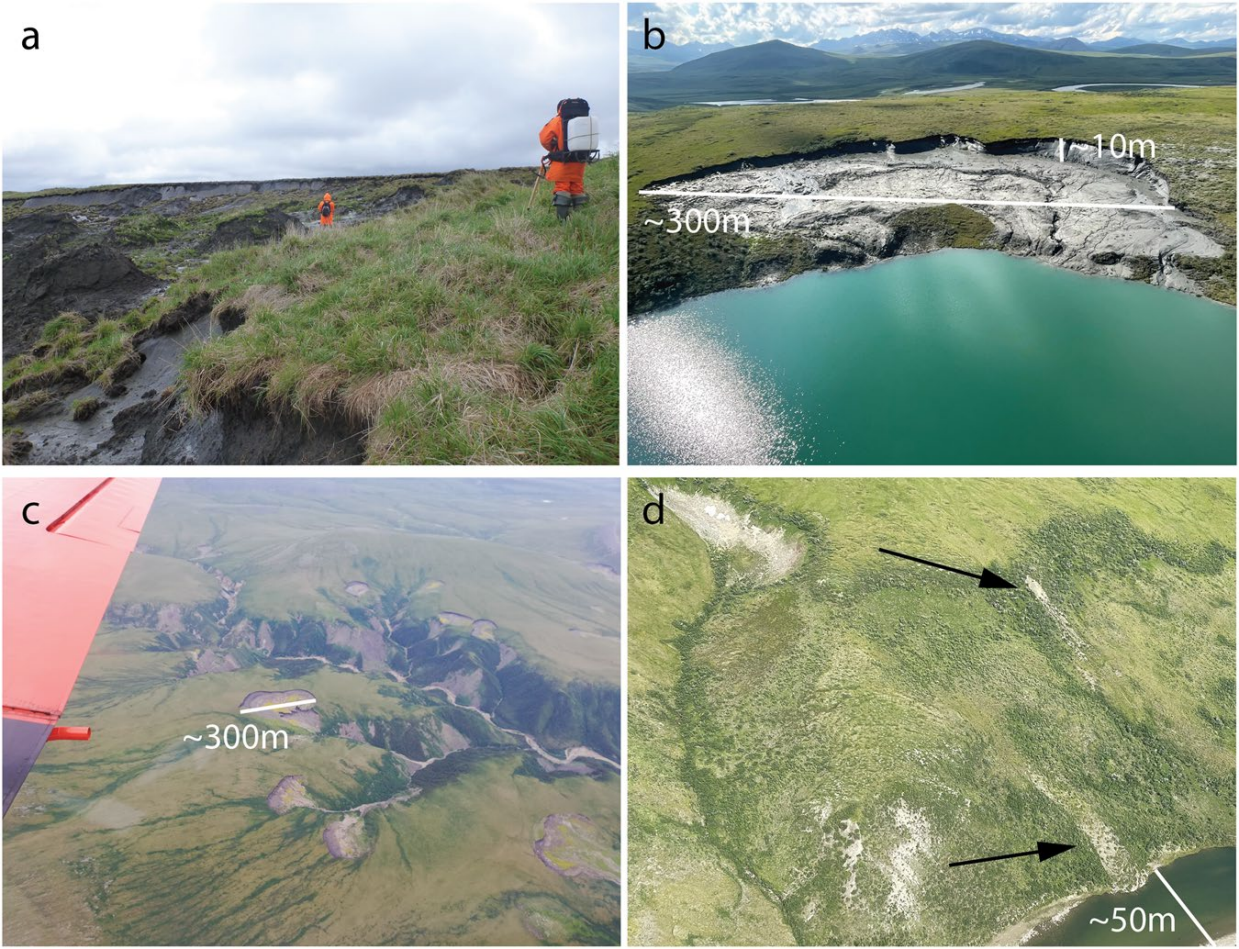
Examples of retrogressive thaw slumps (RTS) and active layer detachment slides. (**a**) Ground view of a coastal elongated/terraced RTS typical for Yedoma ice-rich permafrost on the Bykovsky Peninsula in the Lena Delta Region, northeast Siberia Location: 71.855°N, 129.34°E. Photo: G.Grosse. Persons for scale. (**b**) Oblique aerial photo of an RTS along a lake shore with a notable headwall and scar zone in the Brooks Range in northern Alaska Location: 67.88°N, 156.73°W. Photo: M.J. Lara. (**c**). Oblique aerial photo of multiple large RTS on the Peel Plateau in NW Canada. Location: 68.04°N, 135.62°W. Photo: I.Nitze. (**d**) Oblique aerial photo of active layer detachment slides (marked by arrow) in a previously disturbed hillslope in the Brooks Range in northern Alaska. Photo: M.J.Lara.

Retrogressive Thaw Slumps (RTS) are among the most striking forms of rapid degradation in permafrost regions^1,2^. These mass wasting features, often referred to as hillslope thermokarst, form in inclined or sloping permafrost terrain enabling thaw, meltwater runoff, and the downslope movement of thawed material (see Fig. 1). RTS are triggered by the thawing and collapse of ice-rich ground, which propagates upslope through ice ablation^3^. They consist of distinct morphological landform components, such as a headwall, scar zone, and debris tongue, ranging in size from a few m^2^ up to around 1 km^2^ ^4^. RTS exhibit temporal variability and often display polycyclic dynamics (i.e., recurrence over time)^5,6^ driven by climate change, extreme weather events, and local geomorphological conditions.

They typically occur in regions with ice-cored moraines or ice-rich yedoma permafrost and require sloped terrain to form or to re-initiate, which is often found along shorelines of the sea, lakes or rivers. They were found to occur in various slope ranges with regional differences^2^ e.g. 8 to 12 degrees in the Richardson Mountains in northwest (NW) Canada^7^ or 4 to 15 degrees in the Canadian High Arctic^8^, but typically less than 20 degrees^9^. While these features often exhibit spatial clustering due to specific formation conditions, they remain relatively sparse across the landscape.

Similar to RTS, active layer detachment slides (ALD) are also hillslope thermokarst features. They are shallow permafrost landslides at the base of the active layer (the seasonally thawed layer)^10–12^. These slides often initiate further thaw, which can lead to the formation of RTS^13–15^. In remote sensing imagery, RTS and ALD appear spectrally similar due to the typically dark grey color of bare disturbed ground, but differ in shape and morphometry. However, distinguishing them from other disturbances like landslides or other forms of bare ground can be challenging.

Since the 1980s, the abundance and frequency of RTS have increased across the permafrost region which further accelerated over the past decade^16–21^. The spatial distribution of RTS across the vast permafrost region remains poorly quantified, particularly across large parts of Siberia. While a few publicly available regional datasets exist e.g.^22–25^, our understanding of spatiotemporal hillslope thermokarst dynamics has been largely derived from research concentrated in NW Canada^6,26,27^, NW Siberia^11^, and more recently the Qinghai-Tibetan Plateau (QTP)^25^.

These mapping initiatives are still manually or semi-automatically driven^18,24,28,29^ as RTS can be challenging to detect and delineate properly even for experts^30^. These initiatives have enhanced our understanding of RTS and hillslope thermokarst dynamics, particularly in NW Canada^1,28^, while providing data sources for training more automated detection approaches. In Siberia, ongoing efforts to map and quantify RTS and hillslope thermokarst on Yamal and Gydan Peninsulas^23^ and further sites sites such as Kolguev Island, Novaya Zemlya or Taymyr among others^31^ have also been the focus of similar recent mapping initiatives. However, these inventories based on predominantly manual labeling are labor intensive to create, update, and maintain, which makes them a suitable tool for local to regional scales (particularly in dense RTS clusters), but unfeasible over larger regions or even the entire permafrost region. Automated RTS detection approaches can help to characterize temporally dynamic RTS behavior, while constructing inventories in an unbiased manner.

More automated approaches using remote sensing have been applied to map and monitor RTS on various scales. Over the last decade, machine- or deep learning (DL) techniques using object detection (finding an object) or segmentation (delineating an object) of satellite imagery have become increasingly common methods for detecting and segmenting anthropogenic and natural objects. Nitze *et al*.^32^ also used Landsat trend data to map RTS among other disturbances, but they relied on Landsat imagery with a limited spatial resolution of 30 m, which was often too coarse for many RTS. Similarly, *Runge et al*.^33^ used time series Landsat data analysis with LandTrendr to map RTS-like disturbances across northeastern Siberia. More recent studies^34–37^ focused on object segmentation of RTS using deep learning on higher-resolution data with 3–5 m spatial resolution, such as PlanetScope, or very-high-resolution (VHR) Maxar imagery with 1 m or better spatial resolution. On the QTP, recent advances have been made to map RTS in detail with a hybrid semi-automated hybrid approaches, producing high quality RTS footprints since 2016^25^. However, these sources are commercial datasets and thus costly or only accessible with specific research licenses, which limits their accessibility.

With a second approach, differential elevation data have been used to map elevation and volumetric changes. This includes local studies using photogrammetric methods with high resolution aerial imagery in the Noatak Valley in northern Alaska^38^, the usage of UAV-based multi-temporal local to regional analysis in NW Canada^39^ as well as regional mapping and change analysis based on differential TanDEM-X digital elevation models (DEMs) on a regional scale in northern Siberia^40,41^ or multiple regions across the Arctic^42^. A pan-arctic analysis based on differential ArcticDEM was carried out by *Huang et al*.^43^, who found 2,494 active RTS across the Arctic; however, this underestimates RTS abundance. In a third approach which adds additional data sources to the differential DEM analysis, *Dai et al*.^44^ used ArcticDEM time-series analysis and deep learning to detect large areas undergoing RTS and compiled a panarctic inventory of large RTS (>10,000 m^2^) and synthesis of volumetric and carbon dynamics. *Maier et al*.^45^ used a multi-modal approach where the results of *Xia et al*.^25^ were combined with differential digital elevation models (DEMs), which is relevant for this work as it is a potential avenue to further reduce false positives over rock outcrops or small lakes.

An important aspect in recent research has also been the validation and accuracy assessment of object detection or segmentation with DL. Precision, recall, their harmonic mean (F1 score), and Intersection-over-Union (IoU) are typically used as main metrics, with IoU primarily applied for segmentation task validation. With VHR satellite imagery^36^, tested the applicability of a U-Net deep learning model for segmenting RTS footprints in two sites in the Canadian High Arctic using a fully automated workflow. They achieved F1 scores of 0.75–0.85 on a held-out test set of their training and validation data. Using a similar methodology^37^, employed a U-Net3+ convolutional neural network based on 4-meter Maxar base maps and ArcticDEM elevation data to test an automated approach focused on NW Siberia but also evaluating other sites across the Arctic. They achieved F1 scores of 0.71–0.74, using the same validation scheme. A detailed test and parametrization of various DL architectures used in RTS segmentation with PlanetScope imagery, ArcticDEM-derived elevation and slope data, as well as Landsat trend information to assess spatial transferability and scalability was conducted by *Nitze et al*.^35^. In this study, which used regional cross-validation to test spatial transferability, the models achieved F1 accuracies of 0.25–0.73. Using PlanetScope imagery on the QTP, *Huang et al*.^46^ achieved F1 scores of even 0.85, though in a limited region of 5,500 km^2^. *Heidler et al*.^47^ introduced a novel semi-supervised approach called PixelDINO, which internally generates pseudo-labels and iteratively validates and improves them. Tested on pan-arctic samples using Sentinel-2 data, this method outperformed supervised approaches with F1 scores of 0.46–0.56 compared to 0.37–0.40 for standard supervised models. Across these studies, validation has typically relied on internal datasets split into training, validation, and test sets. While this approach may show good consistency, spatial scaling and transferability present additional challenges. Notably, comparisons with independent datasets or external validation is rare. This lack of external validation may be attributed to the scarcity of compatible datasets, particularly in terms of temporal and spatial scale. Moreover, the high fragmentation of high-quality RTS datasets further hinders cross-study comparisons.

Using common sources for validation and creating larger standardized RTS databases might help to benchmark data products and to upscale processing significantly. First efforts have been taken to provide RTS labels from a wide variety of geographies and RTS types, such as *Nitze et al*.^48^ or the ARTS database^49^. However, as shown in a RTS mapping experiment with multiple contributors of different expertise levels, expert-drawn labels for RTS can vary strongly based on prior expertise, scientific background, and scientific goal of the mapping^30^, which implies that creating proper RTS training and validation datasets is challenging. Furthermore, the use of independent, external datasets for validation likely leads to lower accuracy metrics compared to validation against a subset of the input data, due to different labeling standards and experience, but also other factors like varying temporal overlap, spatial resolution or target geometries (polygons vs. points). Thus, high accuracy metrics presented for a variety of studies can be reasonably expected to be much lower in comparison to external datasets.

In this study, we build upon the work of *Nitze et al*.^35^ to develop a blueprint for a pan-Arctic RTS monitoring system that incorporates regular updates using high-resolution remote sensing, Unet++ Convolutional Neural Networks, and targeted data and image post-processing. Here we present the second revised version (v1.2) of our automated RTS detection dataset called DARTS^50^, which covers RTS hotspots around the pan-arctic permafrost region. The DARTS dataset contains geospatial polygons which represent active RTS or active areas within larger RTS landforms. These polygons are further called features in the manuscript. As DARTS only detects active slumping areas and does not contain inactive vegetated RTS parts it represents an active geomorphological process rather than RTS as a landform, which may also include inactive and ancient parts^3,51,52^.

DARTS covers a total area of ~1.64 million km^2^ with at least one coverage between 2018 and 2023. Our core region encompasses an area of ~900,000 km^2^ and provides at least annual coverage between 2021 and 2023. We here provide a thorough overview of our dataset with used data and methods, as well as validation, the description of possible use cases, and current limitations. Furthermore, we present basic statistics of active RTS abundance and data coverage. The methodology and dataset will be actively maintained and improved in accuracy and spatio-temporal coverage and we envision regular releases in the foreseeable future.

## Data and Methods

### Data

We utilized PlanetScope multi-spectral optical satellite imagery^53^ as our primary data source for extracting the RTS footprints. PlanetScope imagery comprises four spectral bands: Blue, Green, Red, and Near-Infrared, with a ground sampling distance (GSD) ranging from 3.7 to 4.1 meters. The satellites offer a high revisit frequency of less than one day, particularly in high-latitude regions, supported by a fleet of over 180 satellites. Since its launch in 2016, data acquisition frequency has improved, resulting in greater image availability in more recent years. In addition, we incorporated relative elevation and slope data derived from the ArcticDEM mosaic in Version 3.1^54^ and the Landsat Trends (LT) dataset^55,56^. Please find a detailed description below. Our data setup closely follows the methodology outlined in *Nitze et al*.^35^.

We employed two data products from PlanetScope: PlanetScope Scenes (PSScene), which are individual scenes at 3-meter spatial resolution following the original acquisition swaths, and PlanetScopeOrthoTile (PSOrthoTile), which are aggregated data on a gridded footprint with a resolution of 3.125 meters. However, as of 2024, PSOrthoTile products have been discontinued. Initially, we favored PSOrthoTiles for their advantages in data management and organization but have recently transitioned to using PSScene. This mixed input structure is reflected in our output products and metadata. PlanetScope are commercial satellite data. We acquired data initially by directly buying 1 M km^2^ of data and institutional access through University of Illinois. Later we were granted access to NASA’s Commercial Satellite Data Acquisition Program (CSDA) program, which grants NSF affiliated researchers a large quota of free-of-charge access to PlanetScope Data, in our case 10 M km^2^. Data acquisition costs (for academic purposes) were in a range of approximately 0.01–0.10€ / km^2^.

**Fig. 2:**
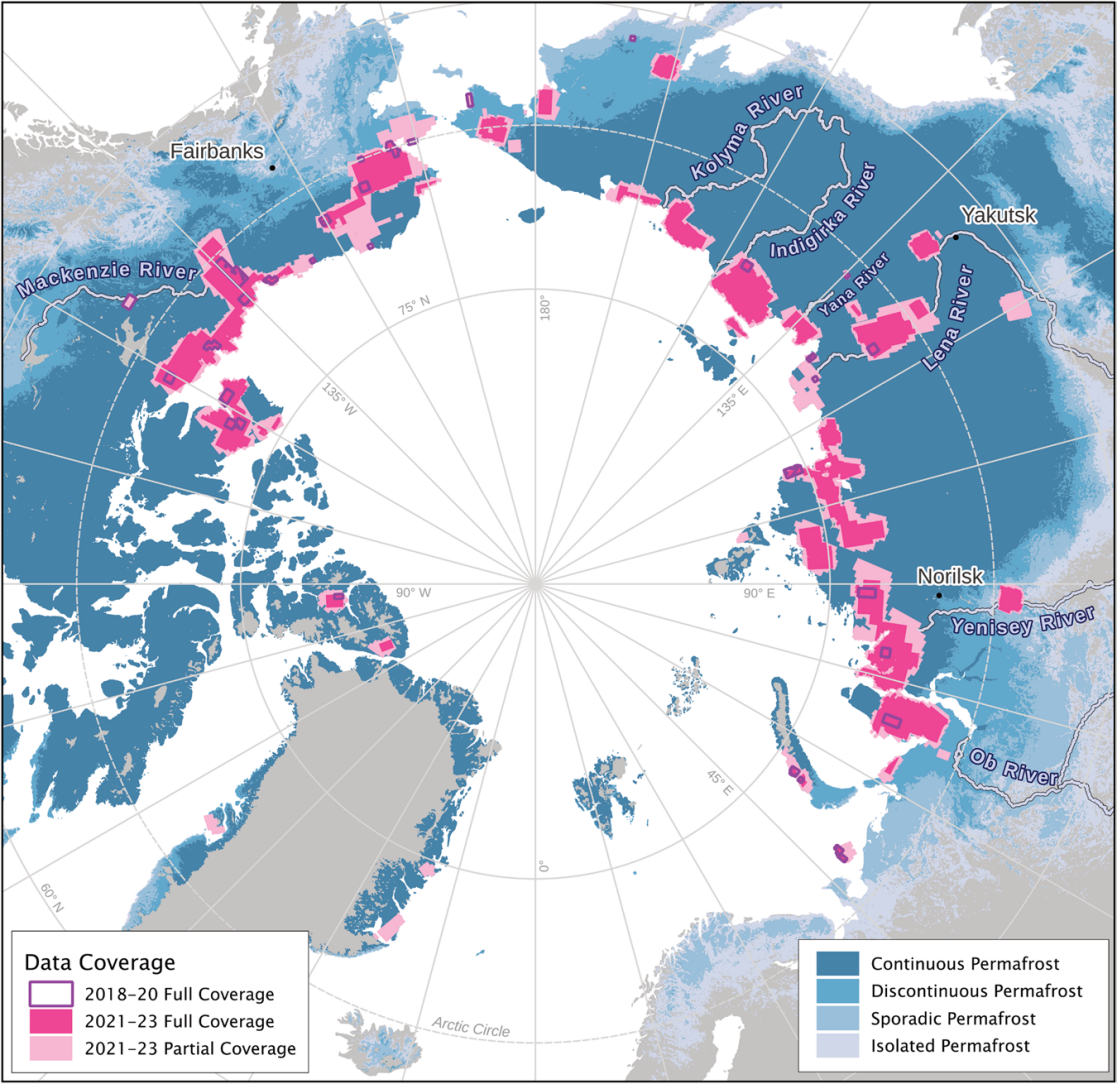
Spatial distribution of DARTS data coverage for core regions in 2018–2023 (dark purple outlines, annual coverage 2021–2023 (dark pink), and partial coverage (at least once) 2021–2023 in light pink. Permafrost extent^70^ is shown in blue shades.

We downloaded data covering a substantial and representative portion of RTS hotspots across the Arctic from 2021 to 2023 (Fig. 2). We began with known hotspot regions based on relevant publications and ongoing RTS research, including Northwest Canada^18,28^, the Yamal and Gydan Peninsulas in Western Siberia^11,23^, and the Taymyr Peninsula in Northern Siberia^40,41^. We then expanded our geographic scope by identifying additional regions of potential RTS activity, using the LT dataset, which visualizes land surface changes over two decades^55^. This allowed us to locate active hillslope thermokarst regions across the Arctic, such as Novaya Zemlya, NW Alaska, and various areas in NE Siberia with RTS in Yedoma ice-rich permafrost, such as Yana-Indigirka and Kolyma Lowlands, which are less documented in the RTS literature. Data for 2021 and 2022 are predominantly from PSOrthoTile, while the 2023 data are mostly PSScene. Gap filling for all years was done with PSScene data.

For selected hotspot regions, such as parts of the Peel Plateau, Banks Island, or East Taymyr, among others, we acquired additional data from 2018–2020, partially at a higher frequency, to extend the time-series for these particularly research-intensive areas, which encompass an area of around 65,000 km^2^ (see Fig. 2). Our data coverage encompasses approximately 1.64 million km^2^ with at least one coverage between 2018 and 2023. Our core region with at least annual coverage between 2021 and 2023 encompasses around 900,000 km^2^. Coverage of individual years (2021–2023) typically covers larger areas, but may not have observations during other years in smaller areas. Overall, we used 17,169 images (PSScenes and PSOrthoTiles) between 2018 and 2023 (Table 1). They cover a total gross area of ~8.35 M km^2^. The majority of images (16,016) fall into our key period between 2021 and 2023 and have a gross coverage of ~7.95 million km^2^ or around 880 billion pixels of raw imagery. For this key period we used 4231 to 5927 images annually, covering a net area (without overlap) of ~1.1 to 1.39 M km^2^ each year. Table 1 lists detailed numbers of data coverage.

**Table 1:** Number and area of input scenes and detected RTS features for both processing levels; Level 1: images scenes and Level 2: annually aggregated data.

Year	Data Coverage			RTS Features			
	Level 1		Level 2	Level 1		Level 2	
	Area [km^2^]	Images [#]	Area [km^2^]	Area [km^2^]	Features [#]	Area [km^2^]	Features [#]
**2018**	121,468	350	65,671	43.82	5,083	32.42	3,539
**2019**	121,673	364	66,324	65.05	5,999	46.74	3,988
**2020**	154,008	439	70,959	81.26	7,417	53.62	4,335
**2021**	2,468,881	5,927	1,197,632	223.79	29,237	146.41	19,733
**2022**	2,586,401	5,858	1,098,728	329.41	35,176	159.44	19,947
**2023**	2,898,801	4,231	1,386,600	266.10	36,537	153.38	22,966
**SUM**	**8,351,232**	**17,169**	**3,885,914**	**1,009.43**	**119,449**	**592.01**	**74,508**
**Union (2018–2023)**	—	—	1,636,692	—	—	286.98	43,572
**Intersect (2021–2023)**	—	—	898,212	—	—	261.71	35,349

We aimed to capture imagery during the peak summer season (July 1 to August 31). When cloud-free data was unavailable, we extended our search into September, which was necessary for far northern sites like the Canadian Archipelago, where coastal fog is less prevalent in late summer. However, low sun angles, large cast shadows, and occasional snow limited usability in September. For data selection, we queried images with less than 20% cloud cover and manually selected data using the QGIS Planet plugin, which offers effective preview and ordering capabilities. Due to limitations in accessible data quotas and insufficient metadata quality, particularly regarding cloud cover estimates, we conducted visual inspections to ensure efficient data usage. For each PlanetScope image we calculated the Normalized Difference Vegetation Index (NDVI)^57^ as a simple feature engineering step, to enhance the vegetation information.

We added derived data from ArcticDEM version 3.1. We calculated a relative elevation, computed as the relative position of the pixel location within a circular kernel with a diameter of 100 m, as used in *Nitze et al*.^35^. We chose the relative elevation to obtain the relative position in the near landscape and to avoid absolute elevation, which are highly variable for RTS. The kernel size was not specifically optimized, but larger kernels generally become computationally more expensive. Furthermore, we calculated the slope values. We preprocessed the DEM derived data in Google Earthengine (GEE)^58^ and downloaded them to local storage and finally created virtual raster tile (*vrt*) mosaics. Additionally, we downloaded the LT dataset, which contains the slope or change rate of Tasseled Cap indices over a 20-year period, based on Landsat data and thus contains basic time-series information of land surface changes. The LT dataset type has been described in^55,56^ and has been used for identifying rapid land surface dynamics in the permafrost regions such as lake changes, wildfires or RTS^32,33,59^. The used Unet++ model architecture is temporally agnostic, thus it can only take one point in time into account in the current version. In the LT time-series information is reduced as the trend of changes to a single image, which allows us to implicitly introduce temporal information to the DL models, which cannot explicitly take multi-temporal data into account. This dataset is available as a public asset in GEE (“users/ingmarnitze/TCTrend_SR_2000–2019_TCVIS”) and covers the period from 2000 to 2019. A more detailed description of auxiliary data is available in^35^. The final input dataset comprises five types of information (satellite imagery, NDVI, relative elevation, slope, and LT) with a total of ten input layers/bands, readily available for the deep learning models (Table 2).

**Table 2:** Overview of input datasets with number of bands, derived dataset if specifically processed, if it was used for the two AI models and the citation of the data source.

Dataset name	Bands [#]	Derived from	*tcvis* model	*notcvis* model	Data source and citation
PlanetScope	4	—	Yes	Yes	Planet Scope data^53^
NDVI	1	PlanetScope	Yes	Yes	Calculated from PlanetScope
Relative elevation	1	ArcticDEM v3.1	Yes	Yes	ArcticDEM^54^
Slope	1	ArcticDEM v3.1	Yes	Yes	See above
Landsat Trends	3	Landsat (5,7,8)	Yes	No	Landsat Trends^56^

## Methods

**Fig. 3:**
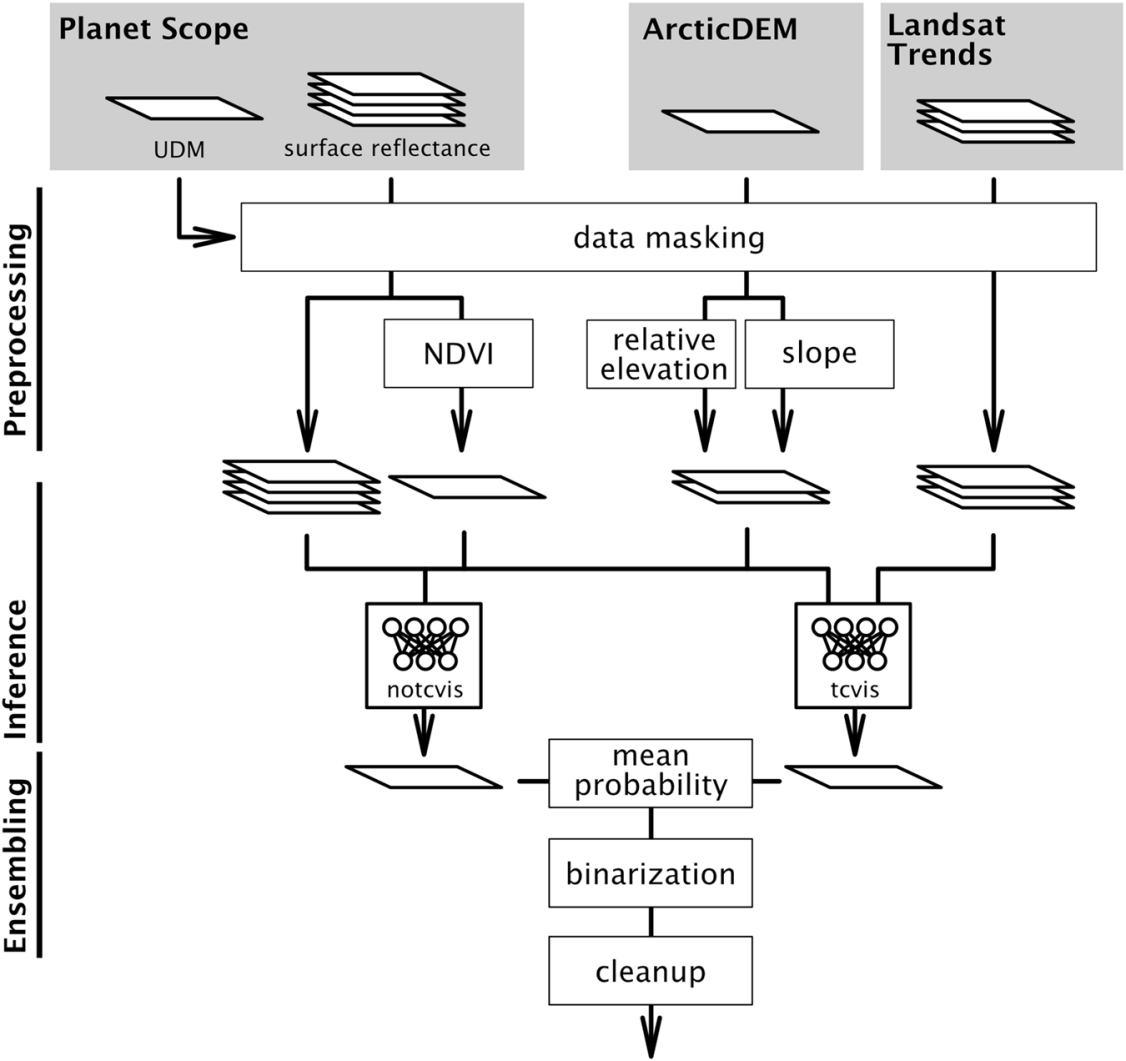
Simplified workflow of RTS dataset inference pipeline with preprocessing, inference, and ensembling stages.

Our deep learning-based dataset processing can be broadly divided into training and inference workflows. These workflows share common steps such as data preprocessing but primarily consist of separate steps. We describe the specific workflows in detail below. The python code for the full data processing workflow is publicly available on github (https://github.com/initze/thaw-slump-segmentation) and published^60^. We utilized PyTorch^61^ together with the Segmentation Models package for the PyTorch library^62^, for the deep learning part of our pipeline. For the data processing part, we used common python geo libraries. This codebase is actively maintained and undergoes continuous improvement. We created the DARTS dataset using our custom thaw-slump-segmentation python package, which has all available functionalities^60^. The schematic workflow for our processing pipeline is shown in Fig. 3.

## Data Preprocessing

### Deep learning model setup

#### Overview

Our model training process consisted of several steps, which we developed iteratively. While the training steps are fundamentally based on *Nitze et al.*^35^, they underwent several further iterations and improvements, such as model architecture selection, hyperparameter tuning, input band selection, addition of new labels, and postprocessing such as filtering end ensembling. The general steps of our pipeline consist of (1) label creation, (2) model training, and (3) model ensembling and data cleaning.

#### Label creation

We followed the labeling procedures outlined in *Nitze et al*.^35^. The labeling process primarily utilized PlanetScope imagery, supplemented by auxiliary datasets such as Landsat Trends^55,56^ to distinguish RTS from stable bare ground like rock outcrops. Furthermore, we used additional very high-resolution datasets such as the ESRI Satellite and Google Satellite basemaps. We implemented an iterative approach for labeling, training, validation, and inference. This process involved training the model(s) and running inference on a larger region after each label iteration, followed by visual inspection and creation of new labels in areas where the previous model underperformed. We completed six iterations in total (001-006), reflected in the available training labels. The initial iterations predominantly focused on positive labels, identifying active RTS regions. In iterations 005 and 006, we introduced more negative samples from regions without RTS to address the high rate of false positives. Our strategic aim was to cover the diverse permafrost landscapes where RTS are or could be present, as well as the general variability of the pan-arctic permafrost region. The final training database contains 3749 features, acquired across 198 image scenes from July 2018 to August 2022 (see Table 3).

**Table 3:** Overview of training datasets with iteration, number of unique image scenes, number of features (RTS), number of images with and without RTS features, and the date range.

	Unique images [#]	Features [#]	Images with RTS features [#]	Images without RTS features [#]	Date range
iteration001	169	2182	149	20	2018-07-02 to 2019-09-28
iteration002	6	743	6	0	2020-07-22 to 2021-07-15
iteration003	4	410	4	0	2020-08-14 to 2021-08-06
iteration004	2	204	2	0	2021-07-21 to 2021-08-06
iteration005	5	141	0	5	2021-07-12 to 2022-07-25
iteration006	12	69	3	9	2022-07-25 to 2022-08-13

The labels, stored as polygons in GeoPackage format, represent the bare soil or scar zone of RTS. We opted to label only the active, unvegetated parts, as this approach is more feasible when using optical satellite images. However, this method may result in lower estimates of the total size of RTS compared to using elevation data as the primary data source and targeting the entire morphological feature like *van der Sluijs et al.*^24^ and as discussed in *Nitze et al.*^30^. The number of features is expected to be higher in actively eroding RTS as the entire landform may consist of multiple active areas, but inactive RTS landforms are omitted. For the labeling process, we primarily worked as a team of two, adhering to internal guidelines and providing mutual feedback. While guidelines are highly recommended and considered best practice, achieving complete label consistency remains challenging^30^. Multiple visual examples of labeled data are shown in^35^.

**Fig. 4:**
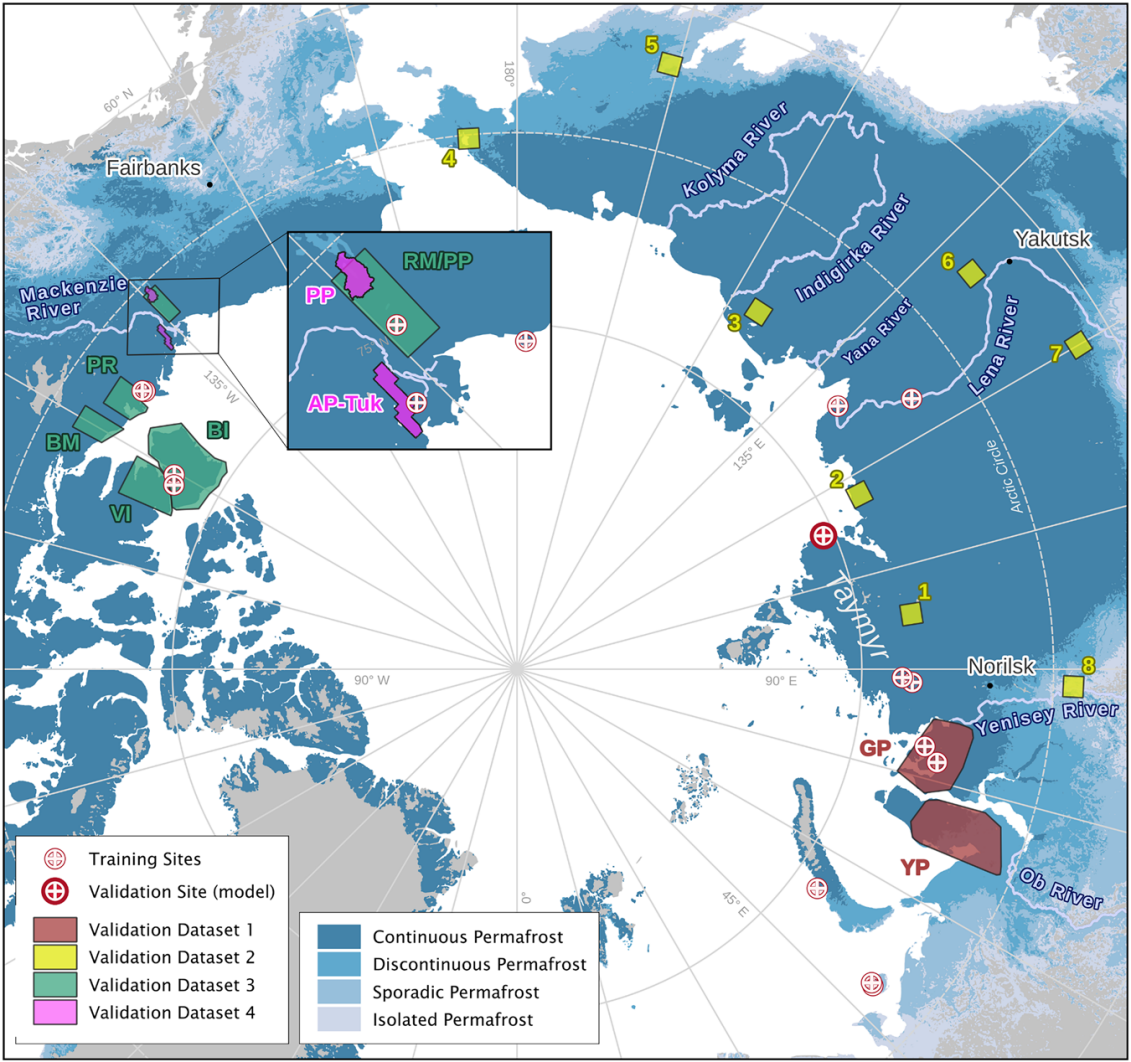
Location of sites where training data was gathered as well as the four dataset validation regions of the dataset validation in NW Siberia (Validation Dataset 1), various regions in Siberia (Validation Dataset 2), the external datasets 3 and 4 by *Lewkowicz*^22^ and *van der Sluijs & Kokelj*^24^ in NW Canada. Model validation was run on a subset of the training dataset (Validation Site (model)).

Training labels are freely available on Zenodo^48^ (https://zenodo.org/records/13935133) and GitHub (https://github.com/initze/ML_training_labels) as polygon vectors in *GeoPackage* format. Footprints of the labeled images are available in the same location. Our training dataset is also part of the ARTS RTS database^49^. Locations of labeled regions are shown in Fig. 4. Details about the training dataset are shown in Table 3.

#### Model training

Our model training was founded on the work of *Nitze et al*.^35^, who performed exhaustive testing of architecture and backbone configurations. Their results showed that Unet++^63^ with a resnet34 backbone performed best. The models from *Nitze et al*.^35^ were based on the first iteration of training labels. We ran six training cycles (iterations) in total, as described above in the *Label* *creation* section.

After the first iteration, we conducted further tuning steps for model depth, learning rate scheduling, tile size, and input band combinations. Our tests included fixed, gamma, and step learning rate schedules. An exponential learning rate scheduler (ExponentialLR) with a gamma of 0.9 and an initial learning rate of 1e-3 proved most effective for our iterative training with pre-trained weights, in iterations 2 to 6. We evaluated tile sizes of 128, 256, 512, 1024, and 2048 pixels, with a ~10% overlap (e.g., 10 pixels for 128-pixel tiles). RTS labels intersecting multiple tiles were cropped. A tile size of 1024 pixels (~3 km) outperformed smaller sizes while remaining more favorable for avoiding GPU memory issues compared to larger tile sizes. We tested model depths of 2 to 6, with a depth of 3 providing reasonably good results while keeping the physical model size manageable for GPU memory. Additionally, we tested different loss functions, with focal loss yielding the best results for our models.

We applied exhaustive augmentation with geometric augmentations, such as *HorizontalFlip*, *VerticalFlip*, and *RandomRotate90*, as well as image quality augmentations, such as *Blur*, *RandomBrightnessContrast*, *MultiplicativeNoise*, and *Cutout* with a 50 percent probability during training. For augmentation we used the *albumentations* python package^64^. During augmentation the number of training patches increased 8fold.

We further tested different input bands. The best configurations predominantly included all data bands/channels (model *tcvis*, n bands = 10). In some instances, leaving out LT, and keeping all other bands (model *notcvis*, n bands = 7) achieved better results. The inclusion of LT helped to identify dynamic areas, but led to false positives in areas with strong landscape change unrelated to RTS, such as along coasts or eroding river shorelines.

For all training and validation runs we used fixed training and validation sets, where the validation set consisted of three scenes in the East Taymyr region in Northern Siberia. We kept the same validation set for all iterations to keep it separate from the training set. For determining the model performance, we used pixelwise IoU and F1 scores of the target class as the key performance metrics, which were implemented by the torchmetrics library^65^.

#### Model ensembling and postprocessing

**Fig. 5:**
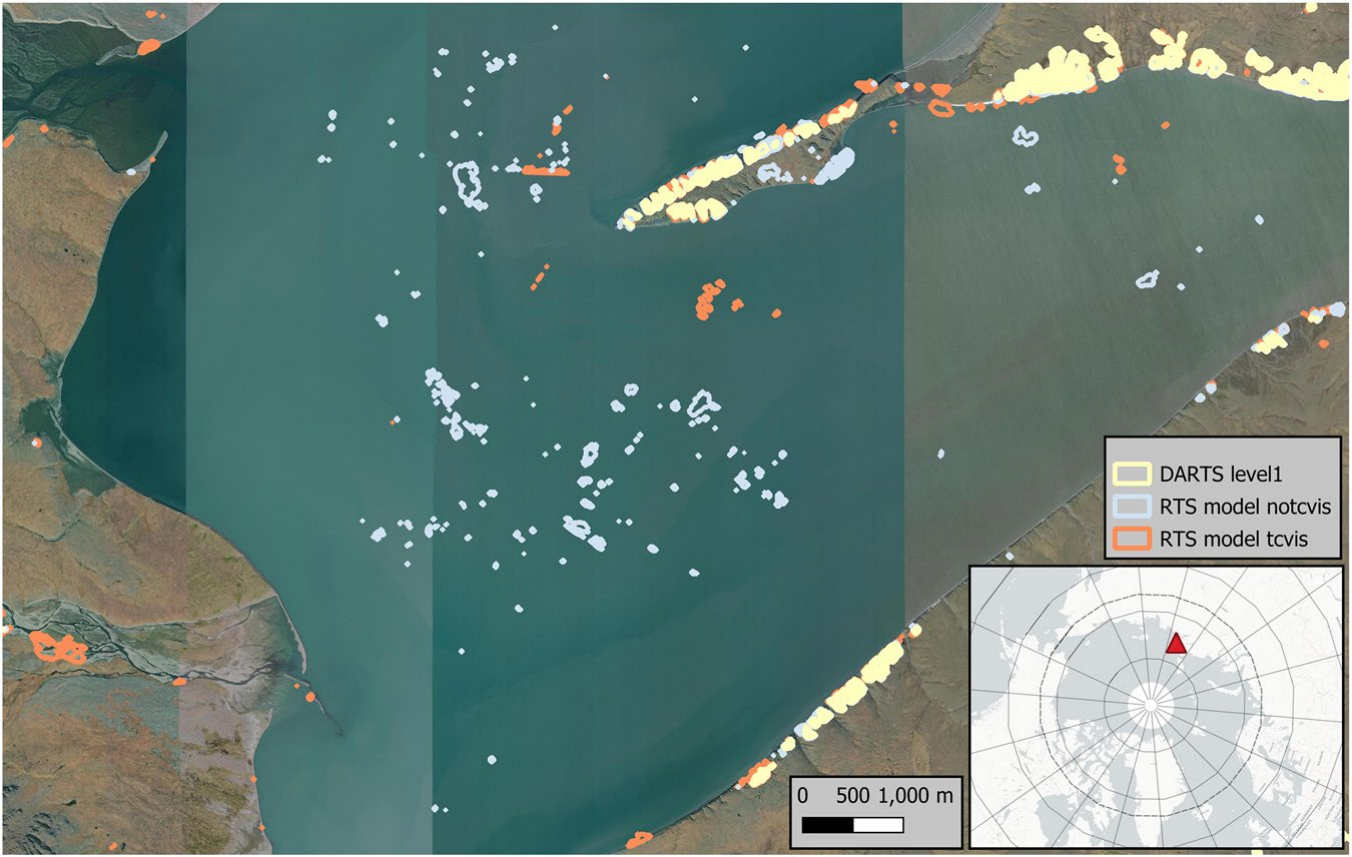
Comparison of raw deep learning model output of *tcvis* (orange) and *notcvis* (light blue) models before cleaning and ensembling versus final DARTS Level 1 dataset, which was ensembled by the raw *tcvis* and *notcvis* model inputs and underwent several cleaning steps. This example is taken from the eastern Taymyr peninsula (75.65°N, 112.97°E). Background Map: *ESRI World Imagery (Sources: Esri, DigitalGlobe, GeoEye, i-cubed, USDA FSA, USGS, AEX, Getmapping, Aerogrid, IGN, IGP, swisstopo, and the GIS User Community)*.

Both model configurations suffered from too many false positives, which were either randomly distributed or overfitted in certain similar regions e.g. close to the image edges and noData, which were typically automatically masked due to clouds. Real RTS were typically detected by both models (Fig. 5). Thus we applied model ensembling and postprocessing at a later stage.

We decided to create a model ensemble where we fused the output pixel-wise probabilities by calculating the mean probability between both model outputs. This helped to minimize noise which was apparent in both individual model outputs. Further we binarized the pixel-wise probability into values 1 for active RTS features and 0 for background, at a threshold of 0.5. This means all contiguous pixels with a per-pixel probability of >=0.5 were aggregated in this binarization step. For the current dataset version, we did not optimize the probability threshold for the binarization. However, we carried out a sensitivity analysis of probability values on the accuracy metrics and dataset size in the dataset validation. On the binarized datasets we deleted all detected active RTS features, which were within 10 pixels (30/31.5 m) of the image border, as the model performed badly in these regions due to typical edge effects. Additionally, we removed all features with a size smaller than 32 pixels, realizing an effective minimum mapping unit of ~300 (288–318) m^2^. After this cleaning step, we vectorized the contiguous objects from the binarized RTS datasets. Furthermore, we used the ESRI 10 m Annual Land Cover (2017–2023) dataset^66^ to clean features over water surfaces. We accessed this dataset from the GEE Community Catalog^67^ through Google Earthengine (GEE) and merged the *Water* and *NoData* mask of the LC map as *Water* as the noData mask was applied over sea water. We then calculated the intersection area of each RTS polygon with the water mask. All Polygons with a fraction higher than 20% *Water* were discarded. This filtering helped to minimize obvious false detections over larger water bodies (Fig. 5). In a final filtering step, we removed all features which intersect with the Sentinel-1/2 derived Arctic Coastal Human Impact dataset v2.0 (SACHI) dataset^68^, which further helped to remove false positives associated with infrastructure, such as roads, railway lines, gravel pits, or urban areas.

### Inference and dataset production

For inference, we developed a highly automated pipeline. Over several years, we downloaded PlanetScope data, initially as PSOrthoTile products and later as PSScene products. Data downloads were typically conducted using the Planet QGIS plugin to visually inspect image quality due to limited data quotas. This visual inspection was more effective at identifying clouds than the standard metadata, helping to avoid data gaps. After the deprecation of the gridded PSOrthoTile products, we managed our internal PlanetScope data with a SpatioTemporal Asset Catalog (STAC), as PSScene data have highly variable data footprints.

For our study area, we processed and downloaded ArcticDEM-derived relative elevation and slope data in large batches using GEE and stored the data locally. The remaining data layers were processed on-the-fly during batch preprocessing. For this step, we created a pipeline that uses a single Planet image scene as the basic data unit to calculate NDVI, download Landsat Trend data from GEE, and automatically clip and resample auxiliary data sources (LT, relative elevation, slope) to match the extent and resolution of the Planet input image. Our processing automatically detects differences between PSScene and OrthoTile images, which vary slightly in pixel size. All input layers are masked where clouds are detected using the pixel-wise usable data mask (udm2) of the input image. This processing step was parallelized to enable scaling.

Finally, all layers were stacked and tiled into 1024 × 1024 pixel patches. We ran inference for both deep learning models, *tcvis* and *notcvis*, which output probabilities (values between 0 and 1), binary raster predictions, and polygon vector predictions of active RTS presence. After model inference on the images, we also executed the ensemble and filtering processes and updated the database with new batches of incoming images. Ultimately, we automatically generated the final product sets with properly assigned metadata and attributes, along with footprint files extracted from our internal STAC catalog.

For processing, we utilized facilities at the Alfred Wegener Institute, including a NVIDIA DGX-A100 node equipped with 8xA100 40GB GPUs. Although the inference process is computationally intensive, the largest bottleneck was data storage and management.

### Validation

To evaluate the accuracy of our dataset, we employed multiple strategies at different stages. Initially, we performed validation of model performance, further called model validation, during the training of our deep learning models against an unseen subset of the training data. This involved pixel-level validation, comparing the specific overlap of feature polygons. We evaluated the pixel-wise accuracy of class labels using standard metrics: precision, recall, F1, and Intersection over Union (IoU). However, this validation only assessed the initial deep learning models (tcvis and notcvis) before ensembling and cleaning. Subsequently, we validated our final dataset against external independent datasets (see details below).

The second validation scheme (*dataset validation*) included two different methodologies; first the manual confirmation of detected features from the DARTS dataset in NW Siberia (dataset validation 1), and second the comparison of DARTS to three different independent datasets with labelled RTS (dataset validation 2–4). This comparison includes manually digitized features across eight sites across Siberia on the one hand, as well as the published RTS datasets by^18,22^ and^24^ on the other hand. The dataset validation was carried out on the object level, thus we compared if our dataset intersected the reference dataset. We refrained from calculating segmentation or pixel level metrics, as both published validation datasets have an earlier baseline (2016 and older) and the comparison of RTS segments is affected by a high uncertainty^30^. All external validation datasets (2–4) are based on different datasets with varying resolutions (see below for more detail).

#### Dataset validation

##### Dataset validation 1: Manual confirmation of features in NW Siberia

As the first strategy, we validated DARTS polygons by manually checking each polygon feature from the year 2023 in the level2 (L2, see *RTS Dataset Description* for details) dataset to determine whether it represents an active part of RTS or if it was falsely detected. From DARTS, we selected a large subset of 3,574 features in NW Siberia on the Yamal and Gydan peninsulas (see Fig. 4). This region covers an approximate area of 105,000 km^2^. We utilized the available ESRI World Imagery Wayback Living Atlas (Esri) and Yandex.Maps (Yandex) satellite basemaps. In particular cases, where the basemaps were of low quality or inconclusive, PlanetScope Scenes were also used. Our dataset mostly overlapped the reference basemaps temporally; however, some differences in temporal coverage may occur. This allowed us to calculate the precision of the dataset for the extensive region of the West Siberian Arctic. Each feature was manually classified as a true positive, false positive, or uncertain. ALD were here considered as true positives, but specifically counted to estimate their fraction of the DARTS dataset. With this strategy, we were able to assess the precision of our dataset. However, we could not evaluate further accuracy metrics, such as recall or F1 score, as there is currently no complete RTS ground truth dataset available yet for this region.

##### Dataset validation 2: Central and Eastern Siberia

To determine variations in model performance across a wide range of permafrost landscapes, we analyzed the model output in eight different areas of Central and Eastern Siberia: Southern Taymyr (#1), Northern Olenek (#2), Chokurdakh (#3), Iultinsky (Chukotka) (#4), Penzhina Bay (Kamchatka) (#5), Southern Verkhoyansk Range (#6), Prilenskoye Plateau (#7), and Turukhansk (#8) (see Fig. 4). These sites cover a total area of 80,000 km^2^, with each site encompassing approximately 10,000 km^2^. The sites contain RTS features to varying degrees, with some regions lacking RTS entirely. This approach also allows us to assess areas without active RTS, which are more typical across the entire permafrost region (see Fig. 4).

Within each of the eight regions, we created ten randomly located squares of 100 km^2^ each. In these subsets, we manually created a reference dataset by generously delineating RTS polygons with a 5-meter buffer based on the following basemap products: we used very-high-resolution ESRI World Imagery for mapping RTSs, while the Arctic Landscape Explorer featuring the Landsat Trend dataset (https://alex.awi.de/), PlanetScope Scenes, Apple Maps, and Yandex Maps were utilized in ambiguous cases. The ESRI Wayback Living Atlas was consulted if none of the aforementioned maps were useful.

We estimated standard validation metrics: precision, recall, and the F1 score of our annual dataset (L2) in these regions. Validation was performed for each year from 2021 to 2023. Our validation was conducted at the object level, testing whether our dataset intersected with the reference dataset. Our reference dataset comprises 272 individual RTS features, of which the majority—235—are located in the four northern regions. After standard validation procedures, we checked the false positives within the large regional subsets of 10,000 km^2^ each for their land cover. We manually assigned each false positive to one of the following classes by visual inspection: Hills/Mountains, River, Sea, Lake, Bare Ground, Vegetation, Periglacial Landform, Other, Uncertain. This effort is intended to better understand the composition of errors to further improve the dataset for future releases.

##### Dataset validation 3: Comparison with reference dataset Lewkowicz 2024

We compared our output data products (L2) with the published RTS point locations in five regions of NW Canada by *Lewkowicz*^22^, namely Banks Island, NW Victoria Island, Paulatuk region, Richardson Mountains /Peel Plateau, and Bluenose Moraine, encompassing a total area of around 154,000 km^2^. These data are evaluated by *Lewkowicz*^18^ (see Fig. 4). This dataset contains the centroids of manually detected RTS based on 30 m resolution Landsat time-series imagery, covering the period from 1984 to 2016 or 2018, compared to our annual L2 dataset from 2021 to 2023. We analyzed the spatial intersection of our dataset, which allows for the calculation of standard accuracy metrics: precision, recall, and F1 score. We analyzed accuracies for the years 2021, 2022, and 2023 of our L2 dataset.

**Fig. 6:**
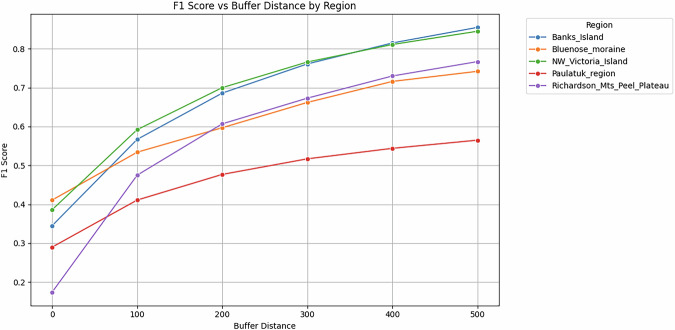
Buffer size analysis for Validation dataset 3 by *van der Sluijs & Kokelj*^22^. Mean F1 score per region depending on buffer size in meters.

Due to the differing temporal periods, and upslope propagation of RTS in this region, we added a buffer to the reference dataset to minimize the effects of spatial inaccuracies and the upslope migration of the active parts of the RTS as the reference dataset only contains centroid points of an earlier period versus more recent active polygons. We tested buffer values from 0 (no Buffer) to 500 m in 100 m increments and found 200 m to be a reasonable value to have a realistic match without artificially inflating accuracy metrics (See Fig. 6). However, this mismatch between both datasets cannot be solved perfectly.

##### Dataset validation 4: Comparison with reference dataset van der Sluijs & Kokelj 2023

We compared our Level 2 (L2) data products with published RTS polygon locations in two regions of NW Canada by *van der Sluijs & Kokelj*^24^: the Peel Plateau (PP) and Anderson Plain-Tuktoyaktuk Coastlands (AP-Tuk), covering a total area of 5,953 km^2^ (see Fig. 4). The reference dataset comprises polygons of manually detected RTS derived from LiDAR and the 2 m resolution ArcticDEM digital elevation model for 2011 and 2016, respectively. It also contains information if slumping activity was observed during the time of observation. We filtered the reference dataset only to active RTS, which matches the approach of DARTS. We analyzed the spatial intersection between DARTS and reference polygons at the object level to determine whether they overlap. This enabled the calculation of standard accuracy metrics: precision, recall, and F1 score. Accuracy assessments were conducted for the years 2021, 2022, and 2023 in our L2 dataset against RTS features from the 2016 validation dataset. Due to the temporal gap between the 2016 reference data and DARTS predictions for 2021–2023 we anticipate a reduction accuracy metrics.

#### Sensitivity of probability values

We analyzed the sensitivity of mean feature probability thresholds in the DARTS dataset versus reference datasets 3 and 4 by iteratively filtering features using minimum thresholds of 0.5, 0.55, 0.6, 0.65, and 0.7. At each threshold, we validated the retained features against datasets 3 and 4, calculating precision, recall, F1 scores, and tracking feature counts to assess the impact of p-value thresholds and reliability of DARTS.

## Data Records

The DARTS dataset in version 1.2 is publicly available on the Arctic Data Center (arcticdata.io) available through: 10.18739/A22B8VD7C.

A short description of sub-datasets and files is provided in Table 4. Detailed descriptions of feature attributes and metadata of all datasets are provided in Table 5 and Table 6.

**Table 4:** Overview of available geospatial dataset files with dataset type, basenames of dataset files, and short description.

Type	Variable name of files	Description
**Features Level 1 (2018–2023)**	*_features_2018–2023_level1	Footprints of RTS features, processed on individual image scenes
**Features Level 2 (2018–2023)**	*_features_2018–2023_level2	Footprints of RTS features, maximum extent aggregated per calendar year
*Coverage Level 1 (2018–2023)*	*_coverage_2018–2023_level1	Coverage of individual image scenes, used for the processing of DARTS Level 1 datasets
*Coverage Level 2 (2018–2023)*	*_coverage_2018–2023_level2	Annual coverage of input images for 2018–2023
*Coverage Union (2018–2023)*	*_coverage_2018–2023_union	Maximum coverage of the dataset with at least one coverage between 2018–2023
*Coverage Intersect (2021–2023)*	*_coverage_2021–2023_intersect	Maximum coverage of the dataset with at least annual coverage between 2021–2023

* Common file basename DARTS_NitzeEtAl_v1-2.

**Table 5:** Detailed overview of DARTS Level 1 dataset attributes, with attribute name, data type, description, and example.

Column Name	Data Type	Description	Example
id	Integer	A unique identifier for each feature in the dataset	0
id_geohash	String	A unique geospatial hash code representing the location of the feature	fqx77bgsy65k
date	String	The date associated with the feature, in YYYY-MM-DD format	2023-08-15
year	Integer	The year extracted from the date column	2023
area_m2	Integer	The area of the feature in square meters	1117
area_ha	Float	The area of the feature in hectares	0.1117
area_km2	Float	The area of the feature in square kilometers	0.001117
image_source	String	The source of the image associated with the feature	PlanetScope
image_id	String	The identifier of the input image image associated with this feature	20230815_191951_90_24af
pval_mean	Float	Mean of AI model per-pixel probability value within the feature	0.531
pval_std	Float	Standard deviation of AI model per-pixel probability value within the feature	0.019
pval_min	Float	Minimum AI model per-pixel probability value within the feature	0.501
pval_max	Float	Maximum AI model per-pixel probability value within the feature	0.566
pval_p25	Float	25th percentile of AI model per-pixel probability values within the feature	0.514
pval_p50	Float	Median (50th percentile) of AI model per-pixel probability values within the feature	0.529
pval_p75	Float	75th percentile of AI model per-pixel probability values within the feature	0.546
DARTS_dataset_version	String	The version of the DARTS dataset used	v1.2
DARTS_dataset_information	String	Additional information related to the DARTS dataset and processing	RTS footprint - individual image (level1)
DARTS_processing_level	String	The processing level of the DARTS dataset	level1
DARTS_AI_model_version	String	The version of the RTS AI model used for processing the data	RTS_v6_ensemble_v3_filterWater
DARTS_AI_model_threshold	String	The lower threshold of the AI model per-pixel probability value	0.5
geometry	Geometry	The geometry column containing spatial data representing the feature’s location	POLYGON ((−68.38536206601792 82.13895427289602…

**Table 6:** Detailed overview of DARTS Level 2 dataset attributes, with attribute name, data type, description, and example.

Column Name	Data Type	Description	Example
id	Integer	A unique identifier for each feature in the dataset	37440
id_geohash	String	A unique geospatial hash code representing the location of the feature	ck8qq7v98kyz
year	Integer	The year extracted from the date column	2022
area_m2	Integer	The area of the feature in square meters	49805
area_ha	Float	The area of the feature in hectares	4.9805
area_km2	Float	The area of the feature in square kilometers	0.049805
image_source	String	The source of the image associated with the feature.	PlanetScope
n_features_level1	Integer	Number of aggregated level1 features.	3
id_geohash_level1	String	Comma separated list of Level 1 features, which are within this level2 feature	ck8qq7v99nzy,ck8qq7v3x7y0,ck8qq7v3gugc
earliest_date	String	The earliest date of observation associated with the feature, in YYYY-MM-DD format.	2022-07-19
latest_date	String	The latest date of observation associated with the feature, in YYYY-MM-DD format.	2022-08-23
n_unique_dates	Integer	Number of unique observation dates	2
pval_mean_of_mean_level1	Float	Mean of pval_mean (mean probability) of aggregated level1 features	0.752
pval_mean_of_max_level1	Float	Mean of pval_max (max probability) of aggregated level1 features	0.903
DARTS_dataset_version	String	The version of the DARTS dataset used	v1.2
DARTS_dataset_information	String	Additional information related to the DARTS dataset and processing	RTS footprint - annually aggregated (level2)
DARTS_processing_level	String	The processing level of the DARTS dataset	level2
DARTS_AI_model_version	String	The version of the RTS AI model used for processing the data	RTS_v6_ensemble_v3_filterWater
DARTS_AI_model_threshold	String	The threshold value used by the RTS AI model	0.5
geometry	Geometry	The geometry column containing spatial data representing the feature’s location	POLYGON ((−123.118 71.433, −123.118 71.433, −123.118 71.433, −123.118 71.43..

For exploring/visualizing and downloading portions of the dataset, please visit the Permafrost Discovery Gateway (https://arcticdata.io/catalog/portals/permafrost).

Training labels for this dataset are available on zenodo^48^ or a publicly accessible github repository https://github.com/initze/ML_training_labels.

### Model checkpoints

https://huggingface.co/ingmarnitze/thaw-slump-segmentation

### RTS dataset description

The datasets are provided in Open Geospatial Consortium (OGC) compliant data formats: GeoPackage and GeoParquet. Both formats consist of single files per dataset. GeoPackage is a widely accepted format for various GIS software, including older versions. We recommend GeoParquet for its significantly faster performance with a high number of features and compact file size; however, compatibility issues may arise with older software versions, such as QGIS or *geopandas*. For reading GeoParquet files it is recommended to use QGIS 3.28 or higher, *geopandas* version 0.14 or higher with the *pyarrow* package for python, and *sf* and *arrow* packages for R in version 4.3 or higher. Recent versions of these applications fully support this format. All files contain polygon geometries. We will further use the term feature to describe geospatial polygon objects, which represent active RTS or active areas within larger RTS landforms.

The DARTS dataset consists of two different processing levels and extensive geospatial coverage files, which provide information on dataset coverage and the boundaries of the input imagery. Level 1 (L1) contains individual footprints of active slumping detected on individual input images. To each feature we assigned a geocoded unique feature ID (id_geohash) using the geometry’s centroid based on the geohash system. We used the *python-geohash* python package to calculate the geohash ids in accuracy L1. Furthermore, we added the source image ID, date, year, area in m^2^, ha, and km^2^, statistics of per-pixel probability values (min, max, mean, standard deviation, percentiles for 25, 50, and 75%) and information about the AI detection model for each feature. Area values were calculated using Lambert Azimuthal Equal-Area (LAEA) projection. For full attribute details please see Table 5.

Level 2 (L2) comprises aggregated data created from L1, representing the maximum RTS extent per calendar year. Overlapping features were dissolved based on the year attribute. The attribute table contains information on the number of observations, the first and last observation dates, and a comma-separated list of unique feature IDs (id_geohash) from L1 data aggregated to create L2 data. Due to this aggregation, L2 data are typically less noisy than L1 data, which are highly dependent on image quality. For RTS with only one annual observation, L1 and L2 geometries are equal. We recommend using L2 data for interannual analysis, while L1 data, despite being noisier, provide higher temporal and spatial resolution for shorter periods. The number of features in L2 is smaller than in L1 due to temporal aggregation (combining multiple observations) and spatial aggregation (merging disconnected L1 features that overlap across time steps). For full attribute details please see Table 6.

The dataset footprints in both processing levels L1 and L2 are provided with coverage files, which provide information on image footprints for L1 and the annual maximum coverage for L2. We further provide the maximum coverage (union), which contains the area, which was at least once covered during the full observation period from 2018–2023. We also provide the region of annual coverage or better (intersect) for our key observation period 2021–2023.

DARTS contains 125,250 features in L1, covering a total area of 1,039.73 km^2^, and 77,405 features in L2, with a total area of 603.34 km^2^ across all years combined. Over the entire observation period from 2018 to 2023, we detected 45,390 unique active RTS within the maximum extent of approximately 1.64 million km^2^ (coverage union), resulting in an affected area of 292.52 km^2^ or 0.179% of the analyzed region. In our core region, which was covered at least annually from 2021 to 2023 and spans around 898,000 km^2^ (Coverage Intersect), we detected 36,447 unique active RTS with a total area of 265.39 km^2^, accounting for 0.03% of the analyzed region (see Table 1).

**Fig. 7:**
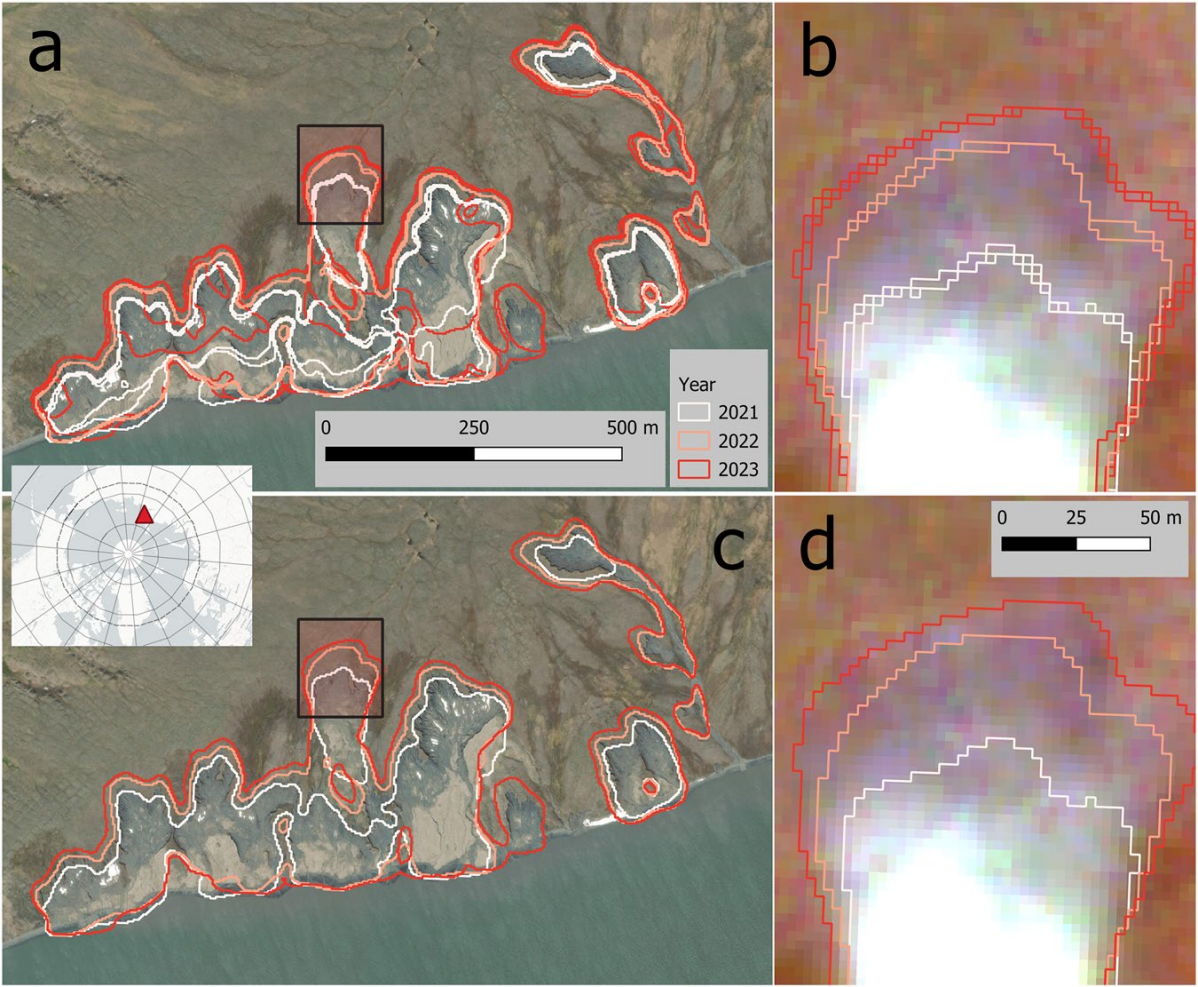
(**a**) DARTS Level 1 (individual observations), (**b**) DARTS Level 1 zoomed to rapidly retreating headwall, note multiple observations per year (2021-08-03, 2021-08-04, 2022-08-14, 2023-07-16, 2023-07-28, 2023-08-08), (**c**) DARTS Level 2 (annually aggregated), (**d**) DARTS Level 2 zoomed to rapidly retreating headwall. East Taymyr, N Siberia, 75.68°N, 113.16°E. Background Maps (**a,c**) *ESRI World Imagery (Sources: Esri, DigitalGlobe, GeoEye, i-cubed, USDA FSA, USGS, AEX, Getmapping, Aerogrid, IGN, IGP, swisstopo, and the GIS User Community);* (**b,d**) PlanetScope imagery from 2023-08-08 © 2025 Planet Labs PBC.

**Fig. 8:**
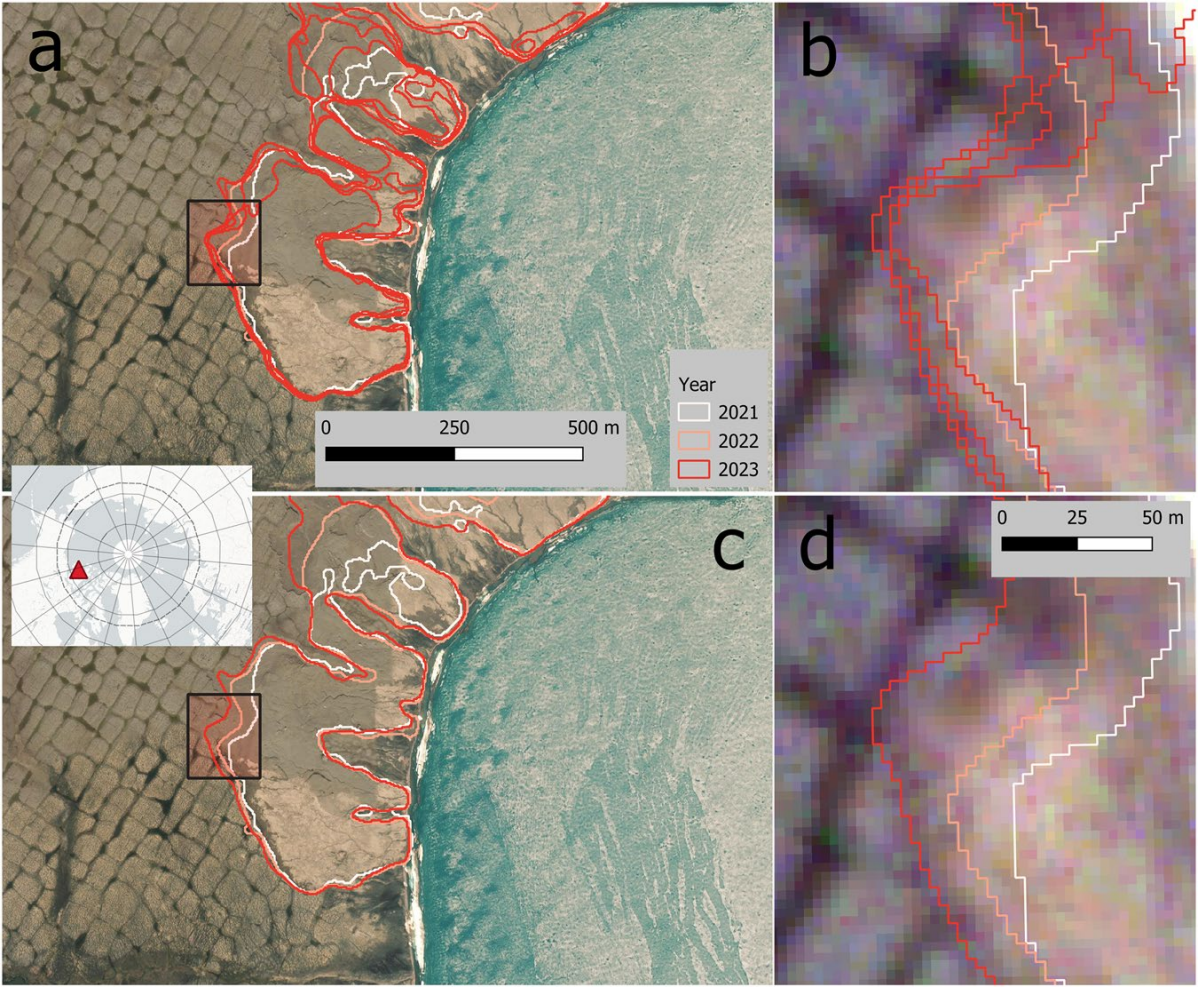
(**a**) DARTS Level 1 (individual observations), (**b**) DARTS Level 1 zoomed to rapidly retreating headwall, note multiple observations per year (2021-09-17, 2022-08-01, 2023-07-28, 2023-07-31), (**c**) DARTS Level 2 (annually aggregated), (**d**) DARTS Level 2 zoomed to rapidly retreating headwall. Banks Island, NW Canada, 72.97°N, 118.13°W. Background Maps (**a,c**): *ESRI World Imagery (Sources: Esri, DigitalGlobe, GeoEye, i-cubed, USDA FSA, USGS, AEX, Getmapping, Aerogrid, IGN, IGP, swisstopo, and the GIS User Community);* (**b,** **d**): PlanetScope imagery from 2023-07-31 © 2025 Planet Labs PBC.

Figures 7 and 8 illustrate the typical expansion pattern upslope, where the headwall retreats over time in our examples from 2018–2023 and 2021–2023. Both examples show a higher number of features for L1. The lower boundaries of the scar zone may be more unstable and variable even within a single year; however, the headwall position is clearly identified. The annual aggregation in L2 removes the fuzziness observed within the scar zone but reduces the temporal resolution to annual values (Fig. 7b/d).

**Fig. 9:**
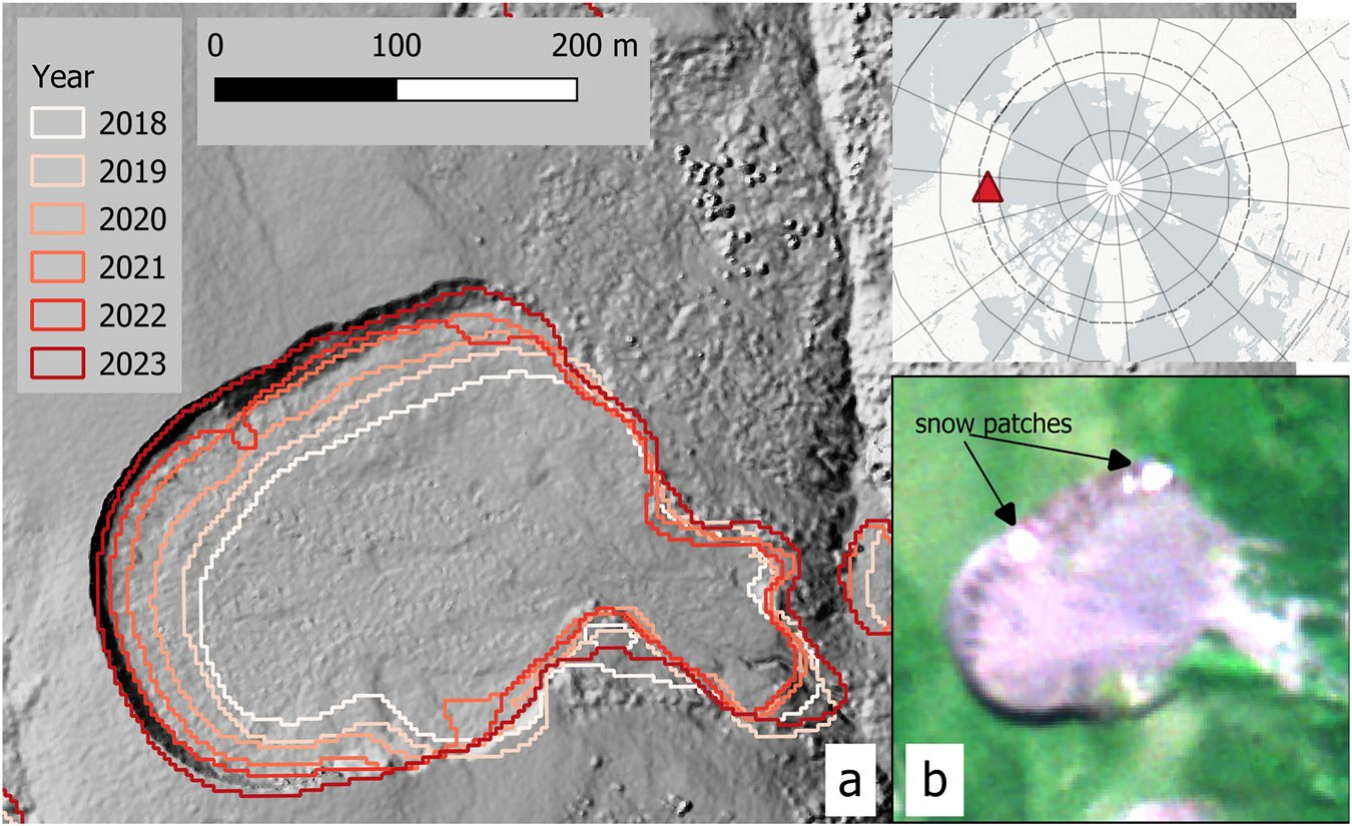
(**a**) Examples of annual DARTS time-series (Level 2) with notable headwall retreat, overlaid on a very high resolution hillshade on the Peel Plateau in NW Canada. Note the protrusion of the 2022 outline along the northern headwall, which was caused by persisting snow patches during the time of observation (2022-07-25), (**b**) PlanetScope image from 2022-07-25 (© 2025 Planet Labs PBC) With snow patches highlighted. 68.21°N, 136.65°W. The Hillshade layer in a) is based on a Structure-from-Motion DSM, acquired on 2023-07-07, of the AWI PermaX 2023 aerial survey campaign.

**Fig. 10:**
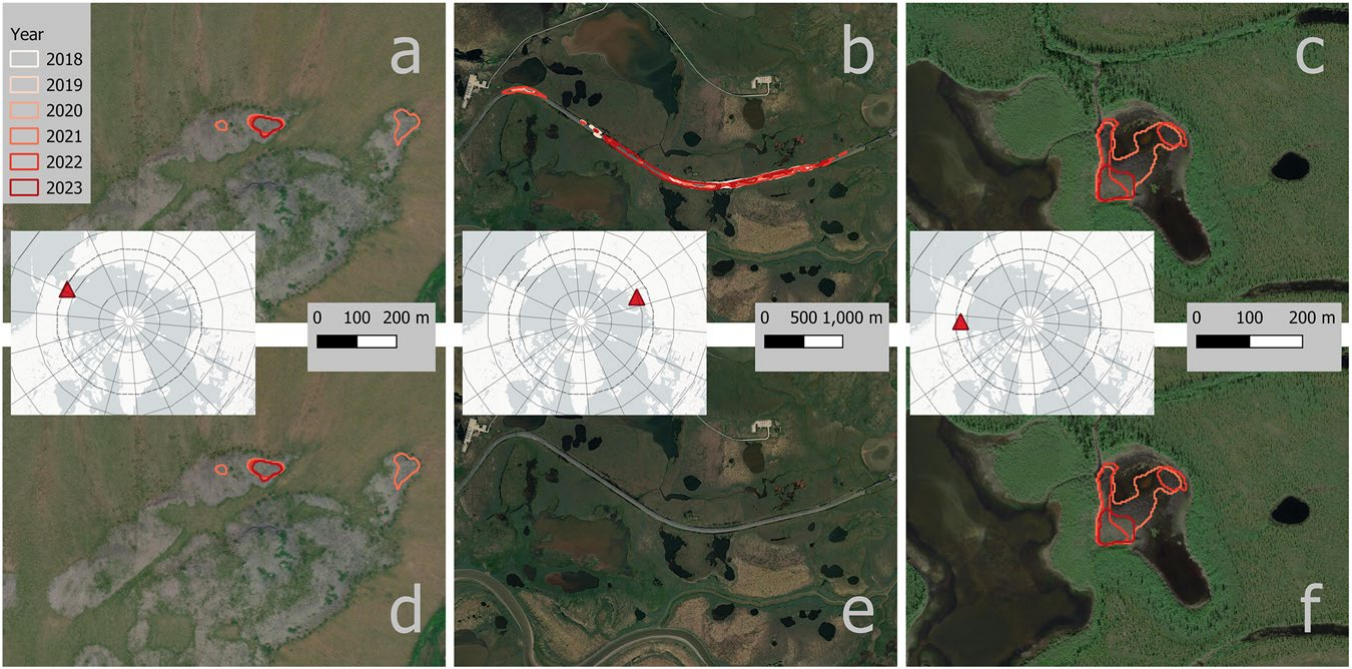
Examples of false detections of RTS of DARTS Level 2 data for version v1.1 (**a**–**c**) and version 1.2 (**d**–**f**). Version v1.2 includes a filtering step to remove infrastructure using the SACHI v2 dataset^68^ and larger water bodies using the ESRI 10 m annual landcover dataset^66^. Example for common false positive locations in (**a,d**) rock outcrops, Brooks Range, Alaska, 67.41°N, 162.41°W; (**b,e**) linear infrastructure, Bovanenkovo, NW Siberia 70.30°N, 68.58°E,; (**c,f**) unsuccessfully filtered small water bodies surrounded by vegetation, Mackenzie Delta NW Canada, 68.13°N, 134.93°W. Background Maps: *ESRI World Imagery (Sources: Esri, DigitalGlobe, GeoEye, i-cubed, USDA FSA, USGS, AEX, Getmapping, Aerogrid, IGN, IGP, swisstopo, and the GIS User Community)*.

Figure 9 compares DARTS L2 data to a high-resolution DSM from July 2023 to demonstrate the detected headwall retreat. Based on visual results, RTS were well detected despite their sparsity, intense polycyclic dynamics, and ambiguity. However, automated processing remains challenging due to specific challenges such as environmental conditions at the time of observation, e.g. snow and ice (See Fig. 9b). Figure 10 illustrates typical or systematic false positive detections, which often occur in small rock outcrops, infrastructure, or small sediment-rich water bodies surrounded by vegetated surfaces. In the current DARTS version v1.2 a large fraction of infrastructure could be automatically removed with the SACHI v2 dataset^68^, while false positive rock outcrops and waterbodies could not be filtered automatically, but will be more thoroughly addressed in future dataset versions.

## Technical Validation

### Model validation

**Fig. 11:**
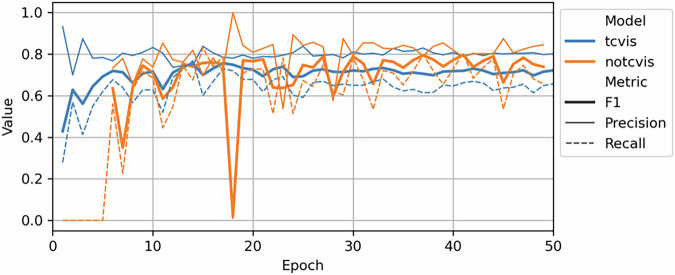
Internal validation metrics of raw tcvis and notcvis deep-learning models.

The model validation set achieved good accuracies, with maximum F1 scores of 0.757 and 0.797, and IoU values of 0.609 and 0.662 for the *tcvis* and *notcvis* models, respectively. The precision and recall of the best models were 0.747 and 0.767 for *tcvis*, and 0.787 and 0.806 for *notcvis*, respectively. The final models were trained and validated on pretrained RTS models from previous generations and converged at around 15 epochs (see Fig. 11).

#### Dataset validation 1: Manual confirmation NW Siberia

Of the 3,574 features present in our dataset, 2,003 were confirmed through manual evaluation. In contrast, 1,616 were identified as false positives, while 42 features remained uncertain, resulting in a precision of 0.560. Due to the absence of a ground truth dataset of this size, we cannot provide recall or F1 metrics for this set. Of the 2,003 true positive features, 161 or 8% were identified as active layer detachment slides.

#### Dataset validation 2: Validation sets Siberia and error types

The manual validation of our dataset revealed varying accuracies across different regions (see Table 7). Overall, the F1 score was 0.323 for 2023 over 0.419 in 2021 to 0.429 in 2022, with a precision of 0.602–0.729 and recall of 0.221–0.304. This precision/recall imbalance suggests an underestimation of features in our dataset compared to the reference datasets. However, for the reference dataset acquisition, image sources with higher spatial resolution were utilized (see Data and Methods).

**Table 7:** Overview of validation results with validation subset, region, precision, recall, and F1 metrics, as well as number of true positive (TP), false positive (FP), and false negative (FN) features.

Val Set	Sub Region	Precision	Recall	F1	Features in the validation set [#]*
Val 1	—	0.560	—	—	2003
Val 2	#1	0.773 ± 0.139	0.392 ± 0.057	0.519 ± 0.078	31
Val 2	#2	0.596 ± 0.271	0.217 ± 0.187	0.310 ± 0.235	35
Val 2	#3	0.992 ± 0.014	0.346 ± 0.076	0.510 ± 0.079	45
Val 2	#4	0.544 ± 0.110	0.253 ± 0.053	0.345 ± 0.071	30
Val 2	#5	0	0	0	0
Val 2	#6	0.306 ± 0.173	0.127 ± 0.047	0.178 ± 0.077	1
Val 2	#7	0	0	0	0
Val 2	#8	0	0	0	0
Val 3	BI	0.737 ± 0.016	0.643 ± 0.038	0.686 ± 0.018	3767*
Val 3	NW VI	0.673 ± 0.013	0.730 ± 0.047	0.700 ± 0.025	1174*
Val 3	PR	0.354 ± 0.057	0.738 ± 0.059	0.478 ± 0.064	123*
Val 3	BM	0.504 ± 0.010	0.737 ± 0.083	0.597 ± 0.022	215*
Val 3	RM / PP	0.552 ± 0.039	0.682 ± 0.065	0.608 ± 0.023	534*
Val 4	PP	0.604 ± 0.067	0.457 ± 0.017	0.519 ± 0.025	272**
Val 4	AP-Tuk	0.794 ± 0.046	0.158 ± 0.008	0.263 ± 0.013	313**

BI: Banks Island, NW VI: Northwest Victoria Island, PR: Paulatuk region, BM: Bluenose Moraine, RM / PP: Richardson Mountains / Peel Plateau, PP: Peel Plateau, AP-Tuk: Anderson Plain - Tuktoyaktuk Coastlands.

*Intersecting DARTS coverage and classified as active in 2016. **Intersecting DARTS coverage and year 2016 as temporal reference.

The variation between regions was significant, with F1 scores ranging from 0 to 0.519 ± 0.078. Regions with a higher abundance of features typically performed better; for example, region #1 and #3 achieved an F1 score of 0.519 ± 0.078 and 0.510 ± 0.079. Conversely, regions with few or no RTS (regions 5–8) exhibited low metrics due to small sample sizes, where a limited number of false positives strongly influenced regional accuracy metrics. Summarized metrics for each subregion are shown in Table 7.

A spatial analysis of 1,513 false positive (FP) features, where land cover type was manually determined, indicated a clear preference for bare ground at 64.4%. This means that nearly two-thirds of the false positives occurred on bare ground, confirming our visual inspection findings. The next most abundant classes were lake and river at 7.87% and 7.17%, respectively, while other classes accounted for less than 5% of the false positive detections (see Table 8).

**Table 8:** Detailed analysis of false positives with manually assigned class type based on validation dataset 2.

Category	Features [#]	Fraction of false positives [%]
Bare Ground	975	64.44
Lake	119	7.87
River	107	7.07
Other	73	4.82
Periglacial Landform	62	4.1
Sea	48	3.17
Uncertain	43	2.84
Vegetation	30	1.98
**SUM**	**1513**	**100**

Examples of false positives are shown in Fig. 10. This figure also highlights the influence of filtering using external datasets, where DARTS v1.1 output without external filters is visualized in the top row (a-c), whereas v1.2 with filters are shown in the lower row (d-f). While the infrastructure was removed successfully (b,e), false positive detections in rock outcrops (a,d) and small waterbodies (c,f) often still persist after filtering.

#### Dataset validation 3: External dataset Lewkowicz 2024

The comparison of our dataset to *Lewkowicz*^22^ in five NW Canadian regions yielded F1 scores of 0.478 ± 0.064 in the Paulatuk region (PR) and up to 0.700 ± 0.0.025 in Northwest Victoria Island (VI), with means and standard deviations calculated over three individual years (2021 to 2023). The F1 metrics exhibit good model performance overall (see Table 7).

Precision ranged from 0.354 ± 0.057 in PR to 0.737 ± 0.016 in Banks Island (BI). Recall was typically higher than precision, ranging from 0.643 ± 0.038 in BI to 0.738 ± 0.059 in PR. The superior recall compared to precision suggests that many features in the reference dataset were successfully detected; however, a slightly higher number of false positives persisted.

Increasing the mean p-value threshold in 0.05 increments improved precision and reduced false positives, but substantially decreased recall, F1 score, and the number of detected features. This trend persisted as the threshold increased further. For example, raising the threshold to 0.55 reduced the number of features by approximately 61.5 ± 3.5%. At the highest tested threshold of 0.7, mean precision ranged from 0.736 in PR to 0.918 in VI, while the number of detected features dropped by 83–95% compared to the original threshold of 0.5 (see Fig. 14(a–c)).

**Fig. 12:**
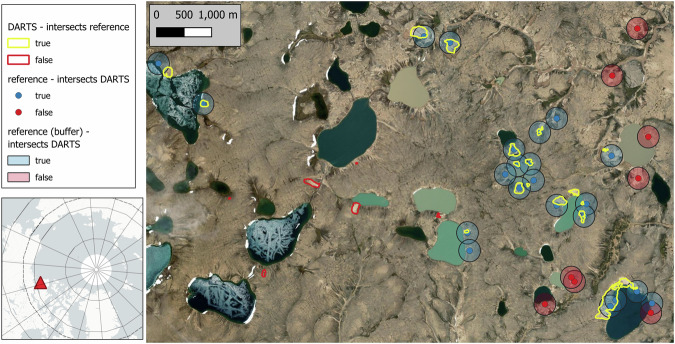
Validation of DARTS dataset with the *Lewkowicz* ^22^ reference dataset on Banks Island (71.68 °N, 122.02 °W). DARTS true positives in yellow and reference true positives in blue (point location + 200 m buffer) with mutual intersection. DARTS and reference false positives, where datasets do not intersect, are indicated in red. Background Maps: *ESRI World Imagery (Sources: Esri, DigitalGlobe, GeoEye, i-cubed, USDA FSA, USGS, AEX, Getmapping, Aerogrid, IGN, IGP, swisstopo, and the GIS User Community)*.

Visual inspection also revealed that some features labeled as FP were actual true features that were either not yet present during the reference dataset’s observation period (1984/1999–2016) (see Fig. 12) or fell below their detection limit. This effect of temporal mismatch is shown in the mean annual performance, with averaged F1 scores of 0.620 ± 0.079, 0.632 ± 0.083, and 0.588 ± 0.112 for 2021, 2022, and 2023, respectively. This might indicate that closer proximity to the reference datasets correlates with better overlap.

#### Dataset validation 4: External dataset van der Sluijs & Kokelj 2023

The comparison of our dataset to *van der* *Sluijs & Kokelj*^24^ in two NW Canadian regions yielded F1 scores between 0.263 ± 0.013 in the Anderson Plain-Tuktoyaktuk Coastlands (AP-Tuk) region and 0.519 ± 0.025 in the Peel Plateau (PP) region, with means and standard deviations calculated over three individual years (2021 to 2023). In both regions precision was notably higher with 0.604 ± 0.067 in PP to 0.794 ± 0.046 in AP-Tuk than recall with 0.158 ± 0.008 in AP-Tuk to 0.457 ± 0.017 in PP. This disparity suggests that most RTS in the DARTS dataset are also present in the reference dataset, but many RTS from the reference dataset were not detected by DARTS, particularly in the AP-Tuk region.

The p-value sensitivity analysis showed patterns similar to those observed in reference dataset 3 (see Fig. 14(d-f). Increasing the p-value threshold improved precision and reduced false positives, but in the AP-Tuk region, this effect only became apparent above a threshold of 0.6. At a threshold of 0.7, both regions achieved perfect precision (1.0). However, this gain in precision came at the cost of sharply reduced recall, F1 score, and the number of remaining DARTS features. Specifically, the number of features decreased by 25–41% at a threshold of 0.55 and by 74–96% at the maximum tested threshold of 0.7. At this highest threshold, F1 scores dropped to just 0.062–0.064, reflecting the very low recall resulting from strict filtering. Due to the strong distribution peak between mean p-values of 0.5 to 0.6, filtering of features, based on mean probabilities, has a strong influence on the number of remaining features and should be used carefully.

**Fig. 13:**
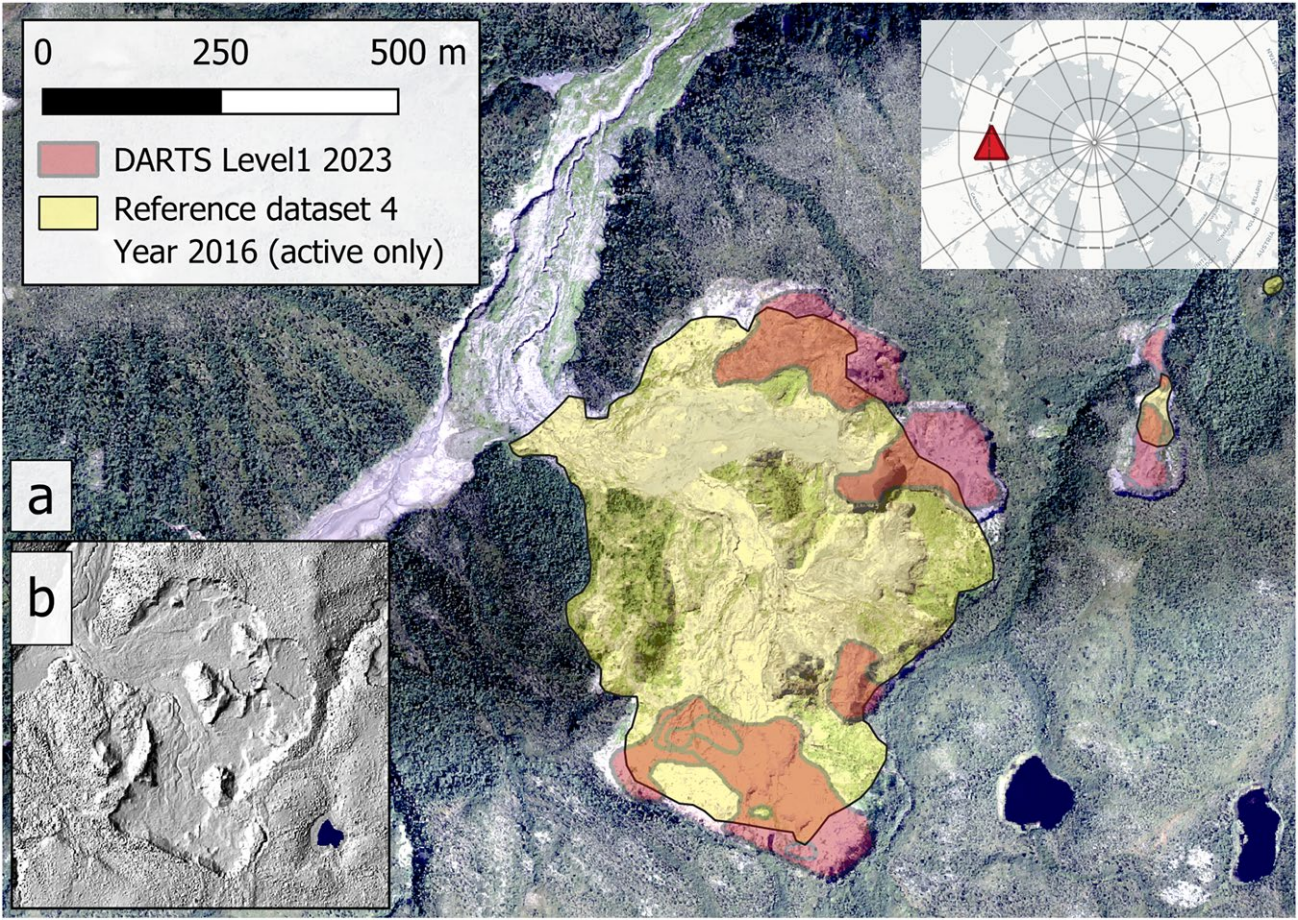
(**a**) Examples of DARTS Level 1 data (2023-07-06, 2023-07-07, 2023-07-28) and reference dataset 4^24^, at the *FM2* megaslump on the Peel Plateau in NW Canada (67.25°N, −135.23°W) overlaid on a very high resolution acquired on 2023-07-15, of the AWI PermaX 2023 aerial survey campaign; (**b**) hillshade of the same location with a focus on the large megaslump processed from the same flight campaign.

As with dataset validation 3, temporal differences between the reference dataset (2016) and DARTS (2021–2023) were apparent. In both regions, all three metrics were generally highest in 2021, slightly decreased in 2022, and further declined in 2023 as the temporal gap widened. The effect of semantic RTS definitions, DARTS detecting active erosion, reference dataset 4 detecting the morphological landform, is highlighted in Fig. 13.

**Fig. 14:**
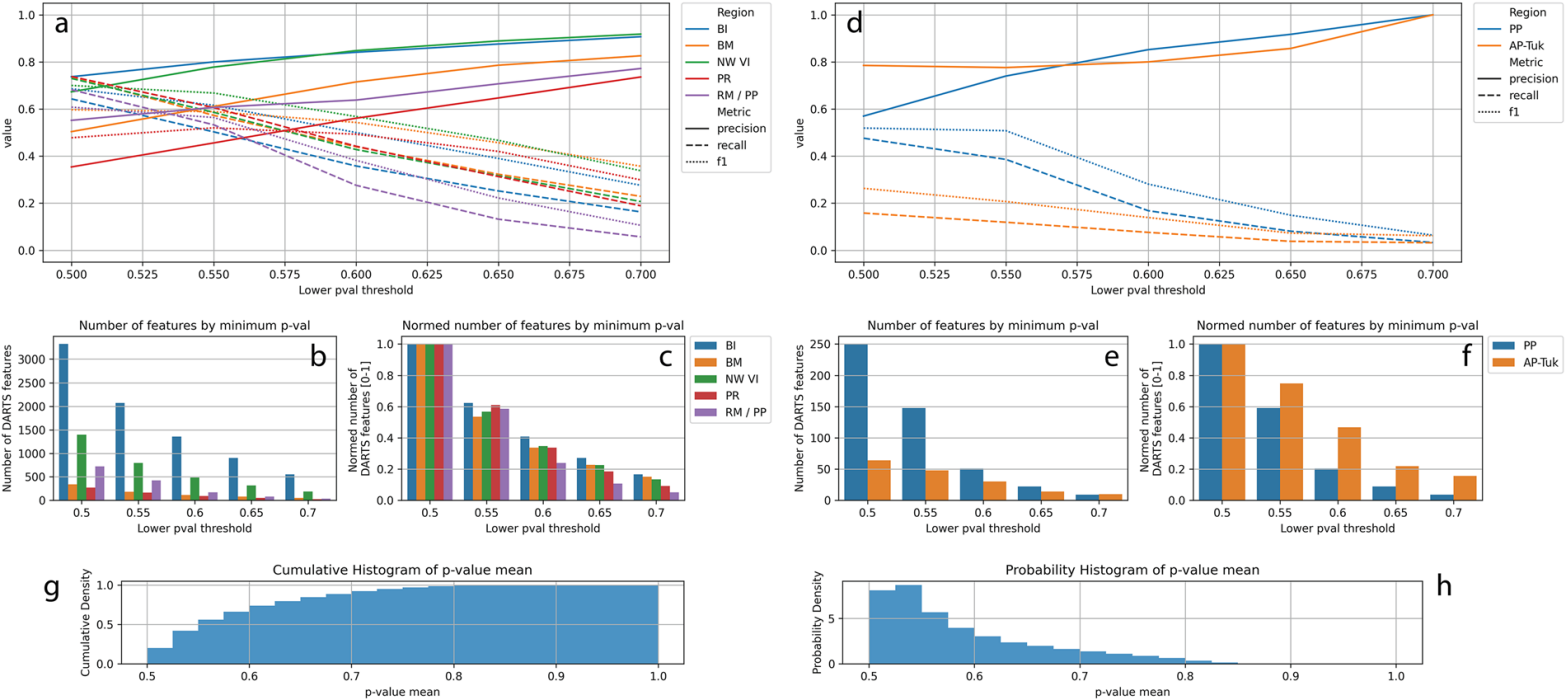
Sensitivity analysis of minimum p-value thresholds of DARTS compared to reference datasets 3 (**a**–**c**) and 4 (**d**–**f**). (**a,d**) as well as cumulative (**g**) and probability densities (**h**) of mean p-values of the complete DARTS Level 1 dataset: Accuracy metrics (precision, recall, F1) per study region as a function of minimum threshold of mean p-values; (**b,c,e,f**): absolute and relative numbers of features depending on minimum threshold of mean p-values.

#### Visual confirmation and spatial patterns

**Fig. 15:**
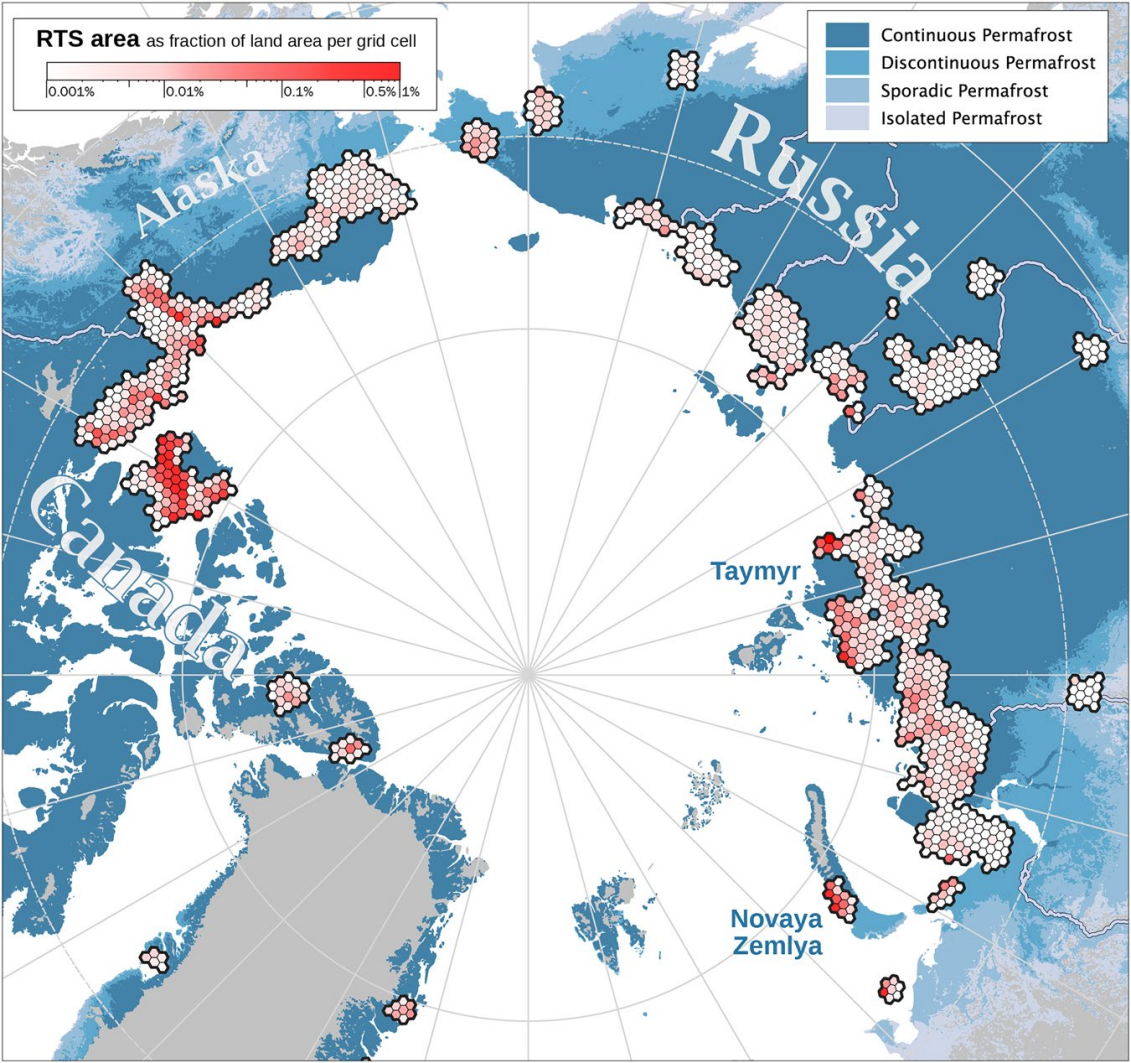
Active RTS area as fraction of land area per grid cell in percent in 2022 based on DARTS Level 2 data. Gridding is based on the H3 grid in level 4 (grid size is ~1000 km^2^ per cell).

**Fig. 16:**
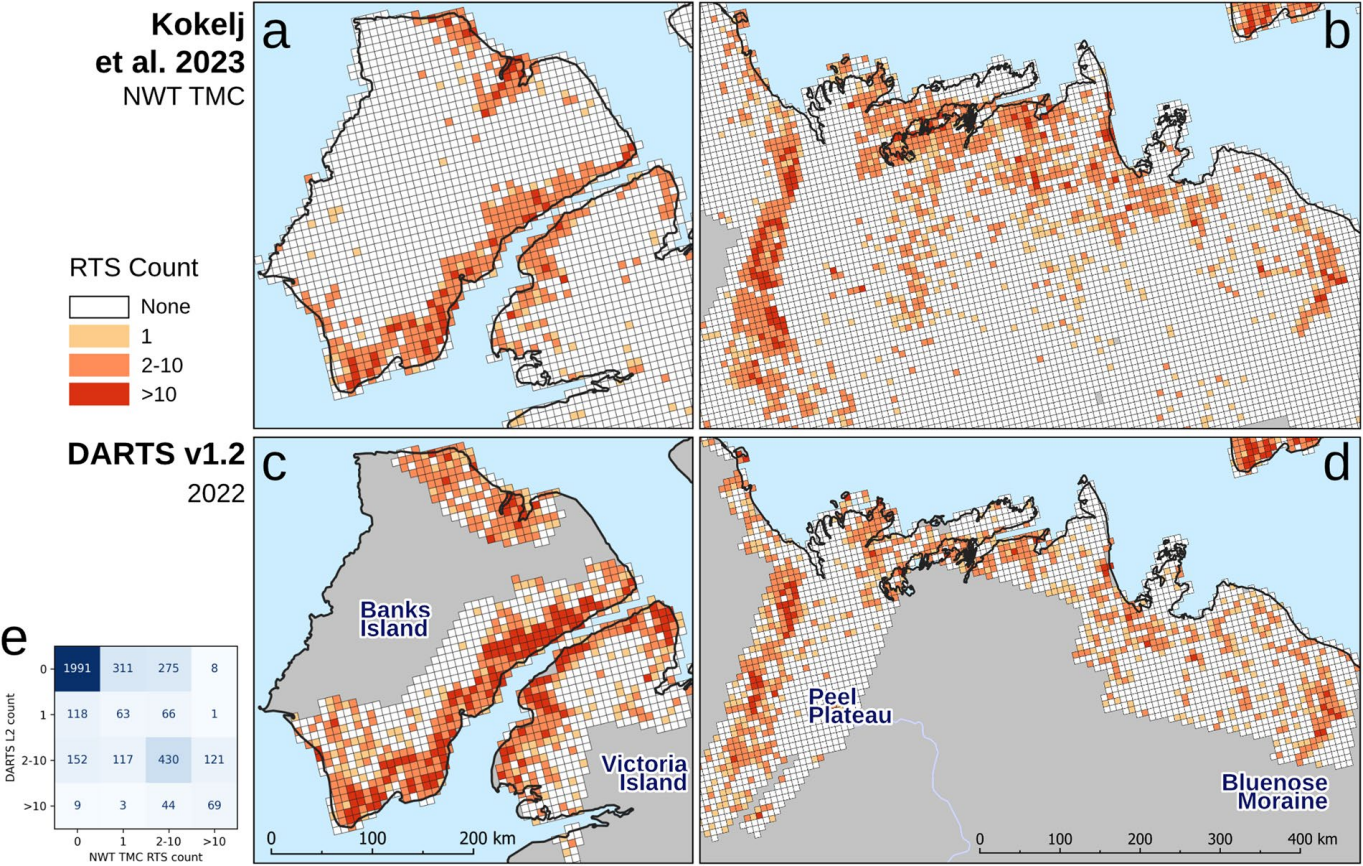
Comparison of the regional Northwest Territories Thermokarst Mapping Collective (NWT TMC) dataset (**a,b**) by *Kokelj et al*.^28^ (dataset in preparation) against the year 2022 subset of our global scale DARTS Level 2 dataset (**c,d**) aggregated to the grid of the reference dataset, and (**e**) confusion matrix of number of RTS per gridcell (n = 3,778) in NW TMC grouping scheme.

Despite the challenges in automatically mapping RTS features, the regional distribution aligns well with other sources and knowledge about the spatial distributions of RTS. Figure 15 illustrates the general patterns of RTS distribution density and area across our pan-arctic research domain, showing the affected RTS area per land area of H3 grid cells (Level 4) in percent. H3 is a hierarchical geospatial hexagonal grid system (Uber Technologies, 2017). DARTS reveals variability in RTS site locations. In Siberia, a hotspot of RTS is evident on the eastern tip of the Taymyr peninsula or the Novaya Zemlya archipelago, matching observation by *Barth et al*.^31^, with widespread occurrences across available regions, typically covering smaller areas. The spatial patterns of active RTS in NW Canada, are generally matching well with data of the Northwest Territories Thermokarst Mapping Collective (NWT TMC) from *Kokelj et al*.^28^ (data publication in prep.), which were kindly provided on request. Areas of high RTS abundance and activity like eastern Banks Island, western Victoria Island, Peel Plateau, and Bluenose Moraine, are well represented in DARTS (Fig. 16). This visual analysis highlights the general distribution patterns, aiding in the identification and analysis of the drivers of RTS presence and activity. The spatial match is also confirmed numerically, as shown in the error matrix in Fig. 16e. Because the reference dataset is aggregated, DARTS cannot be validated at the individual object level. However, to enable comparison, we aggregated the DARTS results to match the feature grouping (0, 1, 2–5, > 10) used for all 3,778 grid cells in the NWT TMC dataset. Nevertheless, both datasets show a similar proportion of cells with detected RTS: the NWT TMC dataset has RTS in 1,193 cells (31.6%), while DARTS has RTS in 1,508 cells (39.9%) out of 3,778, indicating that DARTS contains more RTS features overall. Notably, DARTS includes a higher number of grid cells with exactly one detected feature, as well as cells with ten or more features, compared to the NWT TMC dataset.

## Usage Notes

The DARTS dataset includes feature-specific metadata, such area, dates or probability values. Users can leverage these values to filter the dataset and retain only features with higher confidence. A detailed analysis of the impact of such filtering is provided within this manuscript. We further want to highlight that DARTS contains individual footprints of active RTS or active areas within larger RTS landforms, which might differ to similar RTS datasets based on other data sources, such as differential DEM data. For comparisons between DARTS and other datasets, we recommend using the provided coverage files, which specify the extent of dataset coverage.

## Data Availability

**Processing Code** **Zenodo**^60^**:** Nitze, I., Heidler, K., Küpper, J., & Hölzer, T. (2024). *DARTS RTS AI segmentation code* (Version v0.11.0) [Computer software]. Zenodo. 10.5281/ZENODO.13935921. **Github**: https://github.com/initze/thaw-slump-segmentation. **Training Labels** **Zenodo**^69^**:** Nitze, I., Barth, S., & Küpper, J. (2024). ML training labels (Version v1.0.1) [Computer software]. 10.5281/ZENODO.13935133. **Github**: https://github.com/initze/ML_training_labels. **Model checkpoints** https://huggingface.co/ingmarnitze/thaw-slump-segmentation.
